# Simulations of Proposed Mechanisms of FtsZ-Driven Cell Constriction

**DOI:** 10.1128/JB.00576-20

**Published:** 2021-01-11

**Authors:** Lam T. Nguyen, Catherine M. Oikonomou, Grant J. Jensen

**Affiliations:** a Division of Biology and Biological Engineering, California Institute of Technology, Pasadena, California, USA; b Howard Hughes Medical Institute, Pasadena, California, USA; Université de Montréal

**Keywords:** cell division

## Abstract

To divide, bacteria must constrict their membranes against significant force from turgor pressure. A tubulin homolog, FtsZ, is thought to drive constriction, but how FtsZ filaments might generate constrictive force in the absence of motor proteins is not well understood. There are two predominant models in the field. In one, FtsZ filaments overlap to form complete rings around the circumference of the cell, and attractive forces cause filaments to slide past each other to maximize lateral contact. In the other, filaments exert force on the membrane by a GTP-hydrolysis-induced switch in conformation from straight to bent. Here, we developed software, ZCONSTRICT, for quantitative three-dimensional (3D) simulations of Gram-negative bacterial cell division to test these two models and identify critical conditions required for them to work. We find that the avidity of any kind of lateral interactions quickly halts the sliding of filaments, so a mechanism such as depolymerization or treadmilling is required to sustain constriction by filament sliding. For filament bending, we find that a mechanism such as the presence of a rigid linker is required to constrain bending to within the division plane and maintain the distance observed *in vivo* between the filaments and the membrane. Of these two models, only the filament bending model is consistent with our lab’s recent observation of constriction associated with a single, short FtsZ filament.

**IMPORTANCE** FtsZ is thought to generate constrictive force to divide the cell, possibly via one of two predominant models in the field. In one, FtsZ filaments overlap to form complete rings which constrict as filaments slide past each other to maximize lateral contact. In the other, filaments exert force on the membrane by switching conformation from straight to bent. Here, we developed software, ZCONSTRICT, for three-dimensional (3D) simulations to test these two models. We find that a mechanism such as depolymerization or treadmilling are required to sustain constriction by filament sliding. For filament bending, we find that a mechanism that constrains bending to within the division plane is required to maintain the distance observed *in vivo* between the filaments and the membrane.

## Abstract (precis)

FtsZ is thought to generate constrictive force to divide the cell, possibly via one of two predominant models in the field. In one, FtsZ filaments overlap to form complete rings which constrict as filaments slide past each other to maximize lateral contact.

## INTRODUCTION

To divide, bacterial cells must constrict their cytoplasmic membrane against the large opposing force of internal turgor pressure, but how this is done is not well understood. Rod-shaped bacteria surround their cytoplasmic membrane with a semirigid cell wall composed of long glycan strands running around the cell’s circumference, cross-linked by short peptides into a mesh-like network that provides the cell’s shape template (1–3). Gram-positive bacteria add material to their thick wall in the form of a septum that divides the cell, suggesting that the inward growth of the cell wall pushing on the membrane from outside might be the main force generator. In Gram-negative bacteria, however, we showed recently that a thin cell wall is maintained throughout division (4). Simulating division of this Gram-negative system, we found that cell wall growth alone cannot cause constriction, even with a make-before-break mechanism in which complete hoops of new cell wall are built underneath the existing wall before old peptide bonds are cleaved. Rather, a constrictive force is required to initially relax the pressure on the existing wall, allowing incorporation of new cell wall hoops with a smaller radius (4).

In many eukaryotic cells, a ring formed by actin filaments and myosin motor proteins is responsible for generating a constrictive force to divide cells (5), but motor proteins are absent from bacterial cells. Instead, it has been proposed that the tubulin homolog FtsZ (6) generates the necessary constrictive force at the midcell during division (7, 8). Unfortunately, despite decades of study, how FtsZ might do this is still unclear, and there are strong reasons to doubt all models that have so far been put forward (8).

It is already clear that FtsZ forms filaments that align to the circumference of the cell (9, 10). The distance between the filaments and the membrane is 16 nm (9, 10), but what maintains this distance is unknown. FtsZ filaments are connected to the membrane via FtsA and/or ZipA proteins that bind the C terminus of FtsZ via a flexible linker (11–13). Besides its proposed role as a force generator, FtsZ is also thought to serve as a scaffold for the cell wall synthesis machinery (7, 8). Recently, FtsZ filaments were shown to treadmill around the cell circumference, and it has been suggested that such movement may help distribute the effects of FtsZ uniformly around the cell (14, 15).

There are two predominant conceptual models in the field regarding how FtsZ might generate a constrictive force, filament sliding and filament bending. Observation of bundles of FtsZ filaments *in vitro* (16–25) led to the hypothesis that FtsZ filaments overlap via lateral bonds to form a complete ring which then tightens to constrict the membrane. This was supported by Monte Carlo simulations that showed that lateral attractive interactions between filaments in a closed ring can lead to sliding-induced filament condensation and ring constriction (26). Later, an electron cryotomography (cryo-ET) study showed that complete rings of overlapping FtsZ filaments exist in dividing Caulobacter crescentus and Escherichia coli cells (10), providing experimental support for this filament sliding model. Recently, however, cryo-ET studies from our lab showed that in several species, initial constriction can occur when only a single short FtsZ filament is visible, suggesting that FtsZ can generate a constrictive force without forming a complete ring (27). Alternatively, as FtsZ forms both straight and bent filaments *in vitro* (28–34), it has been proposed that filament bending constricts the membrane. Consistent with this hypothesis, cryo-ET imaging in our lab showed that in dividing C. crescentus cells, FtsZ forms both straight and bent filaments. This result suggested an iterative pinching model in which FtsZ polymerizes into straight filaments which then hydrolyze GTP to bend (pinching the membrane) and then depolymerize to start another cycle (9). In further support of this model, the Erickson lab showed that reconstituted FtsZ can deform liposomes in a process that depends on GTP hydrolysis (35–38).

To explore theoretically how FtsZ filaments might generate a constrictive force in the absence of a motor protein, here, we developed software, ZCONSTRICT, to simulate the two predominant models, namely, filament sliding and filament bending. For each, we identified conditions required for the model to work. For filament sliding, we found that (i) a long-range attractive force between filaments is required to increase lateral contact, (ii) since increasing lateral contact also increases avidity, mechanisms such as filament depolymerization or treadmilling are required to break avidity, and (iii) overlapping filaments have to form a complete ring for sliding to generate ring tension. Exploring the filament bending model we found that (i) a mechanism that causes bending, such as GTP hydrolysis, is required, (ii) bending must be confined within the division plane, for instance, by rigid linkers that prevent filament rolling, and (iii) incomplete rings of filaments must be connected to the cell wall in order for the bending force to overcome the effect of turgor pressure. In the classic back and forth between theory and experiment, we do not claim to show here which, if either, of these models is actually correct, or which assumptions do, in fact, hold in real cells, but rather, we just reveal the necessary conditions for each model to work. It remains for further experiments to reveal what, in fact, happens in actual cells. As the results are three-dimensional (3D) dynamic simulations, best shown in movies rather than static figures, at this point we encourage readers to watch Movie S1 (https://youtu.be/rw88IzRAxEM), which summarizes all the results.

## RESULTS

**FIG 1:**
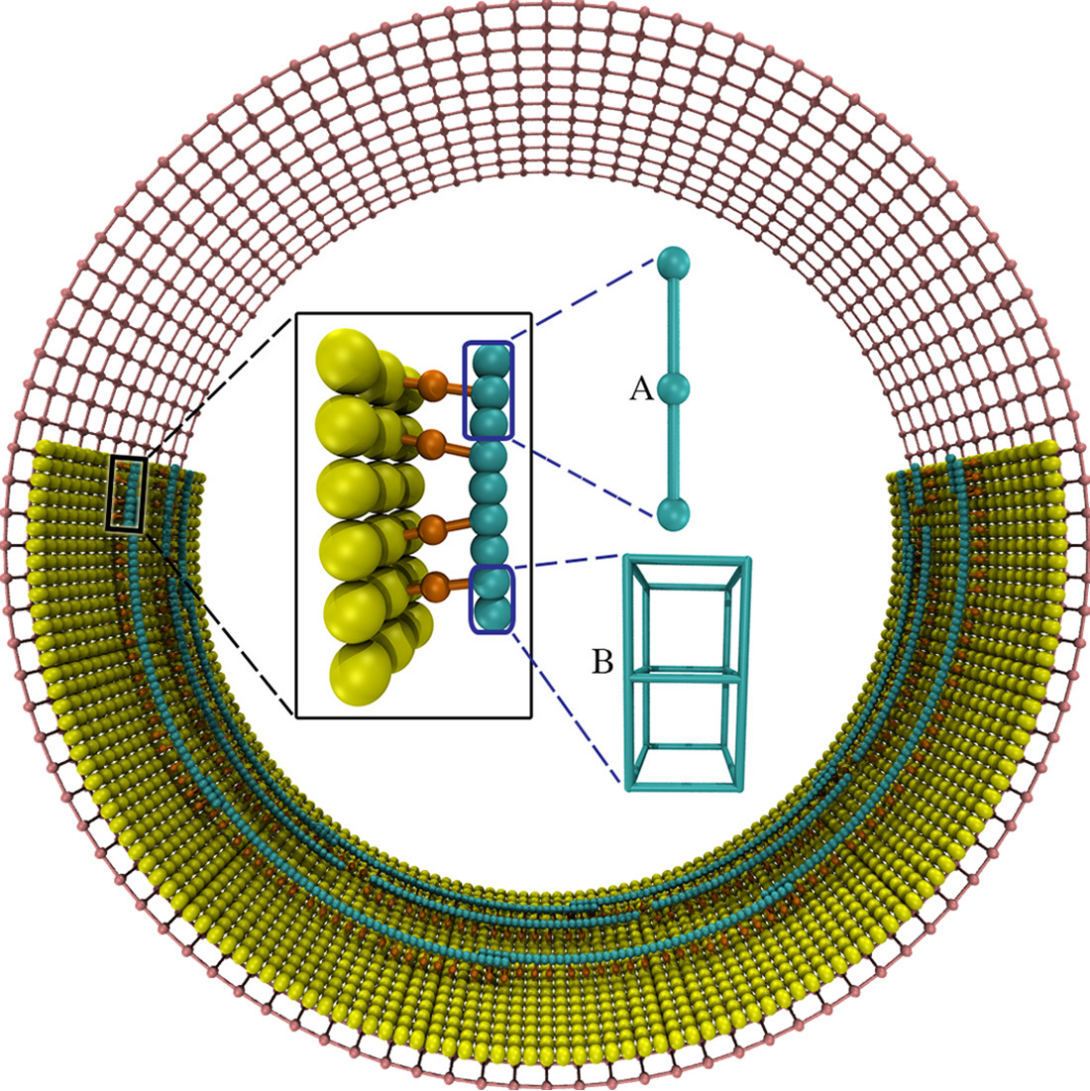
The coarse-grained model. The membrane (yellow) was modeled as a single layer of beads, originally forming a cylinder. Each FtsZ filament (cyan) was modeled as a chain of beads in the filament sliding model (A) or as a chain of cubes in the filament bending model (B). The filament was connected to the membrane via a set of linkers (orange), each composed of two springs (shown as rods) joined at a bead that represents an FtsA or a ZipA protein. The cell wall (pink) was modeled as a grid of beads, originally forming a cylinder surrounding the membrane. Note that the same colors are used for all following figures unless stated otherwise.

We built a coarse-grained model of the system (Fig. 1) in which the membrane was modeled as a sheet of beads, originally forming a cylinder with a radius of 250 nm, consistent with the size of many Gram-negative bacterial cells. FtsZ filaments were initiated in a ring-like arrangement, with a ring radius of 234 nm, separating the filaments 16 nm away from the membrane, a distance that was observed experimentally (9, 10). The filaments were modeled to have different lengths which varied from 88 to 264 nm (20 to 60 beads), resulting in an average length of 176 nm (40 monomers). Note that cryo-ET imaging showed a large variation of the filament length, from ∼100 nm in early stage of constriction to several hundreds of nm in mid- and late stages (9, 10, 27). For the filament sliding model, each FtsZ filament was modeled as a chain of beads connected by springs, with one bead representing one FtsZ monomer, and the filament was originally aligned to the membrane circumference. For the filament bending model, each filament was modeled as a chain of cubes, with each cube representing one FtsZ monomer. To model connections between the filament and the membrane, linkers were composed of two identical springs joined by a bead representing a linking protein, such as FtsA or ZipA. To reflect the flexibility of these linkers, the two springs were allowed to freely rotate around the joining bead without an energy cost. In our model, as the membrane is pulled down by FtsZ filaments, the cell wall moves inward (via insertion of new pepdidoglycan) to fill the gap. For simplicity, we did not model insertion of new cell wall material, but modeled the wall as a grid of beads, originally having a radius of 265 nm, which reduced as the membrane constricted (see Materials and Methods for details). For each model we tested, the key parameters and results are summarized in Table 1.

**TABLE 1:** Summary of key parameters and simulation results of each model

Model	Key parameter(s)	Key result(s)
Core model	FtsZ spring length (*l_z_*) = 4.4 nm; FtsZ spring constant (*k_z_*) = 0.5 nN/nm; FtsZ bending stiffness (*k_θ_*) = 3.8 ⋅ 10^–18^ J; FtsZ-FtsA/ZipA-membrane connection; spring constant (*k_lk_*) = 20 pN/nm; spring length (*l_lk_*) = 8 nm	
Filament sliding		
Adding long-range interaction between filaments	Lennard-Jones potential: depth of potential (ε) = 5 ⋅ 10^–19^ J; zero-potential distance (ρ) = 6.5 nm	Rings failed to constrict due to bundling of filaments
Adding ring separation	Separation distance (*d_s_*) = 20 nm; force constant (*k_s_*) = 50 pN/nm	Filament sliding stopped quickly at high filament overlapping
Adding filament depolymerization	Filament depolymerization rate of 5 beads/sFilament depolymerization rate of 1 bead/s	Rings quickly brokenRings maintained to deep constriction
Replacing filament depolymerization with filament treadmilling	Treadmilling rate of 1 bead/s	Filaments bundled as ring constricted
Filament bending		
Adding GTP hydrolysis-induced bending	Start membrane diameter = 500 nm; intrinsic diameter of the curved filament = 50 nm	Filaments rolled as they bent, forming arcs on the plane of the membrane; implementing treadmilling resulted in filaments moving in circles
Adding circumferentially constrained linkers	Restoring force (to restore alignment of adjacent linkers to the circumferential direction) constant (*k_c_*) = 20 pN/nm	Filaments still rolled partially, forming elliptical tracks
Adding rigid linkers	FtsZ-membrane linker, spring constant (*k_lk_*) = 20 pN/nm and spring length (*l_lk_*) = 16 nm; restoring force (to restore the linker to the radial direction) constant (*k_r_*) = 3 pN/nm; restoring force (to restore the bending of filaments to the division plane) constant (*k*_dp_) = 20 pN/nm	Bent and treadmilling filaments could pull the membrane down
Implementing reverse bending	Start membrane diameter = 500 nm; intrinsic diameter of the curved filament = 250 nm	Reversely bent and treadmilling filaments caused membrane constriction
Implementing single bent filament	Filament preferred curvature of 1/32 nm^−1^	Single treadmilling filament caused constriction

### Filament sliding model.

**FIG 2:**
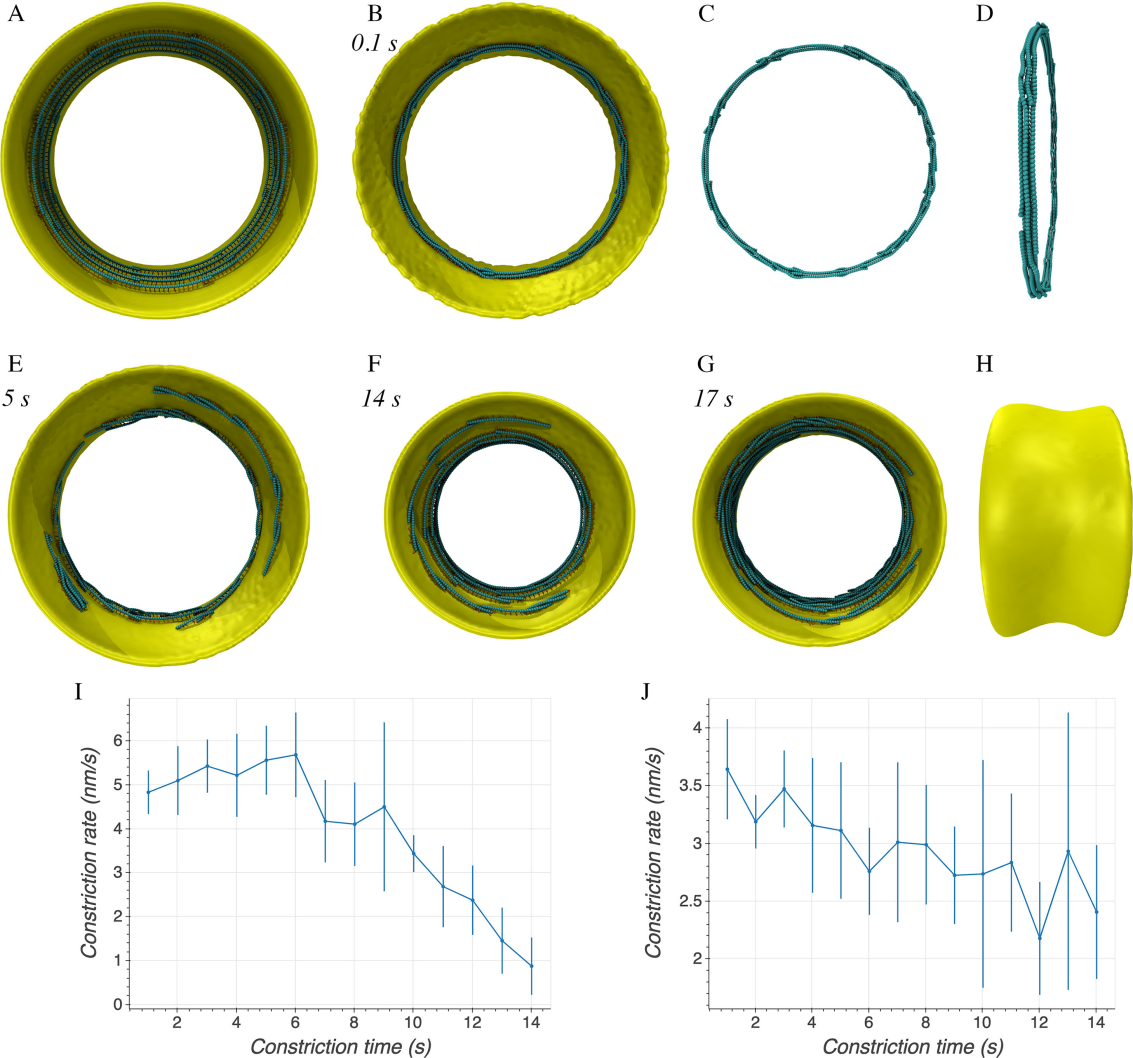
Simulation results of the filament sliding model. Italic font indicates simulation times. (A) Axial view of the initial system in which the FtsZ filaments overlapped to form complete rings. (B) Implementing a Lennard-Jones potential interaction between the filaments resulted in formation of a bundle of rings. (C and D) Axial (C) and side (D) views of the filament bundle in panel B. (E) Implementing depolymerization with a rate above the critical rate quickly resulted in loss of ring integrity. (F) With a depolymerization rate slower than *r_c_*, deep constriction occurred and rings broke much more slowly. (G) Removing depolymerization and implementing treadmilling also resulted in a deep constriction. (H) Side view of panel G. (I and J) Time series of the constriction rate with the simulation conditions were the same as those in panels F and G, respectively. The average was calculated over four simulations. Error bars indicate standard deviation.

**(i) Long-range interaction between filaments.** We reasoned that for filament sliding to generate a constrictive force on the membrane, at least two conditions must be present. First, a long-range attractive force has to exist between the filaments so that when Brownian motion results momentarily in further overlap, that further overlap is energetically favored. Second, overlapping filaments have to form a complete ring, or else no tension would be generated (note that we later verified this when we observed that broken rings fail to constrict the membrane.) We then constructed filaments that overlapped to form complete rings, separating adjacent rings by a distance of 20 nm (Fig. 2A). We then implemented a long-range Lennard-Jones-like potential between the beads on different filaments (see Materials and Methods, “Filament sliding model,” for details). Simulations of this system showed that the rings failed to constrict the membrane; instead, they simply collapsed into a twisted multifilament bundle, and avidity prevented any further filament sliding (Fig. 2B to D).

**(ii) Ring separation.** Since no cryo-ET studies of dividing cells have ever shown twisted multifilament bundles (instead, FtsZ filaments are always seen lying side by side in flat ribbons parallel to the membrane) (9, 10, 27), something more complex must be happening in real cells. One possibility is that there could be proteins, or even the disordered C-terminal tail (39), that hold FtsZ filaments apart. To implement this hypothesis in our simulations, since the filaments were initially arranged in separate rings, we added a repulsive force between filaments of different rings at distances closer than 20 nm (see Materials and Methods for details). As a result, bundling was prevented, but filament sliding still stopped quickly (see Fig. S1 in the supplemental material). To investigate the cause, we calculated the free energy of two laterally interacting filaments as a function of their overlap (Fig. S2). We found that as the overlap increased, so did the energy barrier to further movement, so avidity prevents further sliding.

**(iii) Filament depolymerization.** One way such avidity could be overcome is if filaments were constantly depolymerizing (Fig. S2). In this case, the filament sliding rate would be limited by the depolymerization rate. But because turgor pressure pushes the membrane outward against the cell wall, and the filaments are tethered to the membrane, the filament sliding rate would also be limited by the rate of inward cell wall growth. In order to explore these relationships, we introduced filament depolymerization by removing beads one by one at the minus end with a rate of 5 beads/s and set the maximum inward growth rate of the cell wall at a high value, 100 nm/s. In this condition, rings were quickly broken, indicating that depolymerization was faster than sliding (Fig. 2E). To understand this, we noted that before the rings broke, the wall moved inward with a rate (∼ν_radial_) ∼ 14 nm/s (Fig. S3), equivalent to a circumference reduction rate of ν*_cir_* = 2πν_radial_ = 88 nm/s. As the average length of the filaments was set to be 176 nm (40 monomers), complete rings of 234 nm radius were composed of at least 9 filaments. The filament sliding rate was therefore ν_cir_/9 ∼ 10 nm/s. Thus, depolymerization rates higher than *r_c_* = 2.3 beads/s (given that the distance between adjacent beads was *l_z_* = 4.4 nm) would be predicted to result in ring breakage. We therefore reduced the depolymerization rate to just 1 bead/s, and this fixed the problem, resulting in deep constriction and only infrequent ring breakage (Fig. 2F). Interestingly, the constriction rate gradually slowed over time (Fig. 2I), likely due to both the occasional loss of ring integrity and the increasing resistance to further membrane bending. To confirm that the constriction rate is limited by the filament depolymerization rate, we set the maximum inward growth rate of the cell wall to be even higher, 1,000 nm/s, to be sure the rate of cell wall growth would not be the limiting factor, and then tested various depolymerization rates. As expected, faster depolymerization rates resulted in faster constriction (Fig. S4).

Because for asymmetric proteins like FtsZ, interactions between parallel filaments must be different than interactions between antiparallel filaments, we also ran simulations in which the strength of the interactions between parallel or antiparallel filaments was halved. Constriction was slower in both cases, and the second scenario also led to frequent ring breakage (Fig. S5, left), indicating that ring integrity depends more on interactions between antiparallel filaments.

**(iv) Filament treadmilling.** Since it has been recently found that FtsZ filaments treadmill around the cell circumference (14, 15), we speculated that like depolymerization, treadmilling might also prevent avidity-induced seizure. To explore this, we implemented filament treadmilling, i.e., adding beads one by one at the plus end and removing beads one by one at the minus end with the same rate, assigning the treadmilling direction of each filament randomly in accordance with the experimental observation that FtsZ filaments in cells are seen to treadmill in both directions (14, 15). As expected, simulations with a treadmilling rate of 1 bead/s resulted in constriction (Fig. 2G and H) with the constriction rate reducing over time (Fig. 2J). However, we also observed the formation of bundles of filaments (Fig. S6). Detailed analysis revealed different scenarios for how filaments treadmill with respect to one another (Fig. S7). If two adjacent filaments are antiparallel, as they first pass each other they will have a brief period of short overlap in which sliding could occur. Later, further treadmilling will increase the overlap and stop the sliding through avidity. Once the heads pass the others’ tail, treadmilling will begin to reduce their overlap, until filament sliding is again possible briefly (until there is no more overlap). If two adjacent filaments are parallel, they treadmill in the same direction, and the degree of overlap does not change, so treadmilling does not facilitate filament sliding. As described above, we again tested how varying the strength of interactions between parallel filaments and those between antiparallel filaments affected constriction. Similar to simulations of filament depolymerization, reducing the potential either for the parallel interactions or the antiparallel interactions decreased the constriction rate, while the latter led to ring breakage (Fig. S5, right).

### Filament bending model.

To simulate bending, we represented each FtsZ monomer as a cube with vertices connected by springs. Note that in most figures of this model, the cubes are still shown as beads for simplicity.

**FIG 3:**
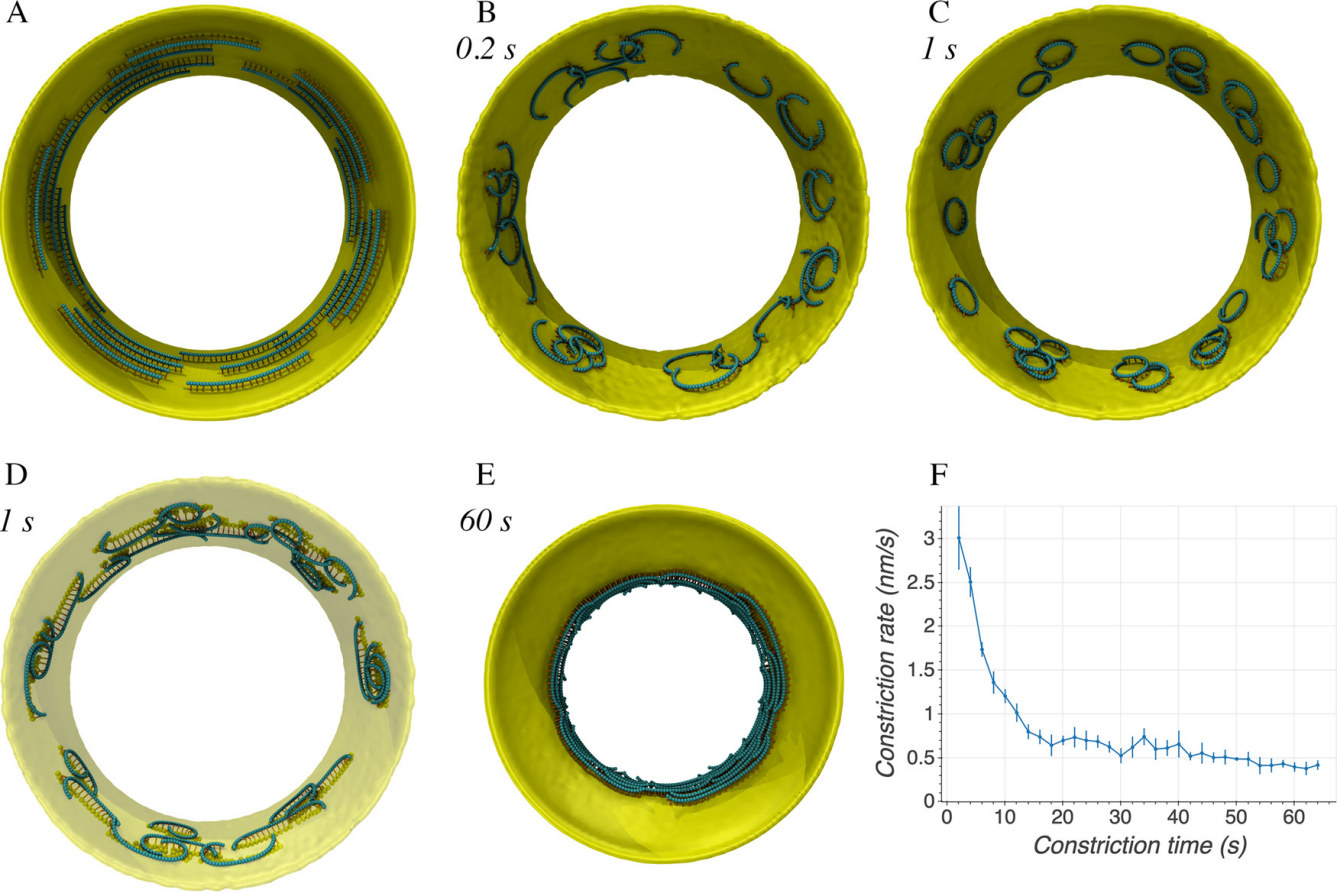
Simulation results of the filament bending model. Italic font indicates simulation times. (A) The initial system with FtsZ filaments in the GTP state running circumferentially. (B) After the filaments were switched to the GDP state, they did not constrict the membrane but, instead, rolled to bend on the plane of the membrane. (C) Implementing treadmilling did not prevent rolling, but filaments treadmilled in circles on the plane of the membrane. (D) Aligning membrane beads (yellow) connected to the same filament to the circumferential direction did not prevent filament rolling but only stretched the filament circles into a more elliptical shape. Note that, except for beads connected to filaments, the rest of the membrane (visualized as a surface) is shown with low opacity. (E) The presence of rigid linkers that prevented rolling allowed filaments to exert force on the membrane. Treadmilling resulted in uniform membrane constriction. (F) Time series of the constriction rate with the simulation conditions the same as in panel E. The average was calculated over four simulations. Error bars indicate standard deviation.

**(i) GTP hydrolysis-induced bending.** FtsZ filaments are thought to be straight when in the GTP-bound state and switch to a bent conformation upon GTP hydrolysis (8). It is likely that both GTP- and GDP-bound states coexist in real cells, but for simplicity in our simulations, all filaments were initiated in the GTP state (all springs of equal length) and switched to the GDP state once the simulation started by setting the relaxed length of the two springs on the C-terminal side to be longer than the that on N-terminal side (Fig. S8 and S9; see Materials and Methods, “Filament bending model”). To test whether filament bending can deform the membrane, we started with a membrane of diameter 500 nm (Fig. 3A). We set the curvature of the filaments to relax at a diameter of 50 nm (10 times more curved than the initial membrane), which is approximately twice the size of FtsZ minirings, the smallest rings that have been observed *in vitro* (28). In our simulations, filament bending did not lead to membrane deformation; instead, filaments rolled as they bent, eventually forming arcs on the plane of the membrane (Fig. 3B). This topology is more energetically favorable and is consistent with the prediction by Erickson et al. (8). For filament bending to deform the membrane, the cell must therefore have a mechanism to keep the bending in the division plane.

To test whether filament rolling was still energetically favored with dynamic filaments, we introduced filament treadmilling with a rate of up to 14 beads/s. The simulations showed that treadmilling did not prevent rolling but, rather, caused the filaments to form treadmilling circles (Fig. 3C), similar to experimental observations of reconstituted FtsZ filaments on a flat membrane (40).

**(ii) Circumferentially constrained linkers.** Searching for mechanisms to prevent filament rolling, we wondered whether connecting filaments to the cell wall might reduce rolling since glycan strands could provide a circumferential scaffold in real cells. To test this hypothesis in as simple a way as possible, we constrained all the membrane beads connected to the same filament to be aligned circumferentially (see Materials and Methods, “Circumferentially constrained linkers,” for details). Because both the filaments and linkers were flexible, partial rolling still occurred, causing the filaments to treadmill in elliptical tracks (Fig. 3D). In addition, we observed that filaments were pulled closer to the membrane (Fig. S10B and C) than real FtsZ filaments are (∼16 nm, as seen by cryo-ET [Fig. S10D]) (9, 10).

**(iii) Rigid linkers.** It makes sense that flexible linkers cannot prevent filament rolling or maintain a regular distance between the filaments and the membrane, so we hypothesize that there is some kind of more rigid linker inside real cells. The C-terminal peptide of FtsZ has been shown to be flexible, but it may become more rigid as it binds other proteins *in vivo*, for example FtsA, ZipA, and ZapABCDE (11–13, 41–48). Arguing against this, it has been shown that FtsZs linker sequences from different organisms can be swapped, and the cell still divides normally (10, 47, 48), so it remains quite unclear what could form a rigid linker *in vivo*. Nevertheless, in the spirit of discovering the essential conditions required for each model to work, we implemented rigid linkers between the filament and the membrane. To maintain the filament-membrane distance, we first modified the linker (originally modeled as two springs joined at a central bead) to be a single spring (Fig. S11) (see Materials and Methods, “Rigid linkers,” for details). We then constrained this linker to the radial direction of the membrane. We assumed this linker bound the folded domain of FtsZ tightly and therefore prevented filament rolling, which in turn constrains bending to within the division plane. Simulations of this system showed that the filaments could now pull the membrane down. Adding filament treadmilling, membrane constriction occurred uniformly (Fig. 3E). Similar to the filament sliding model, the constriction rate decreased wilth time (Fig. 3F). We reasoned this was because (i) as the membrane radius decreased, the difference in curvature between the membrane and the filament and therefore the constriction force decreased and (ii) the bending resistance from the more curved membrane increased.

**FIG 4:**
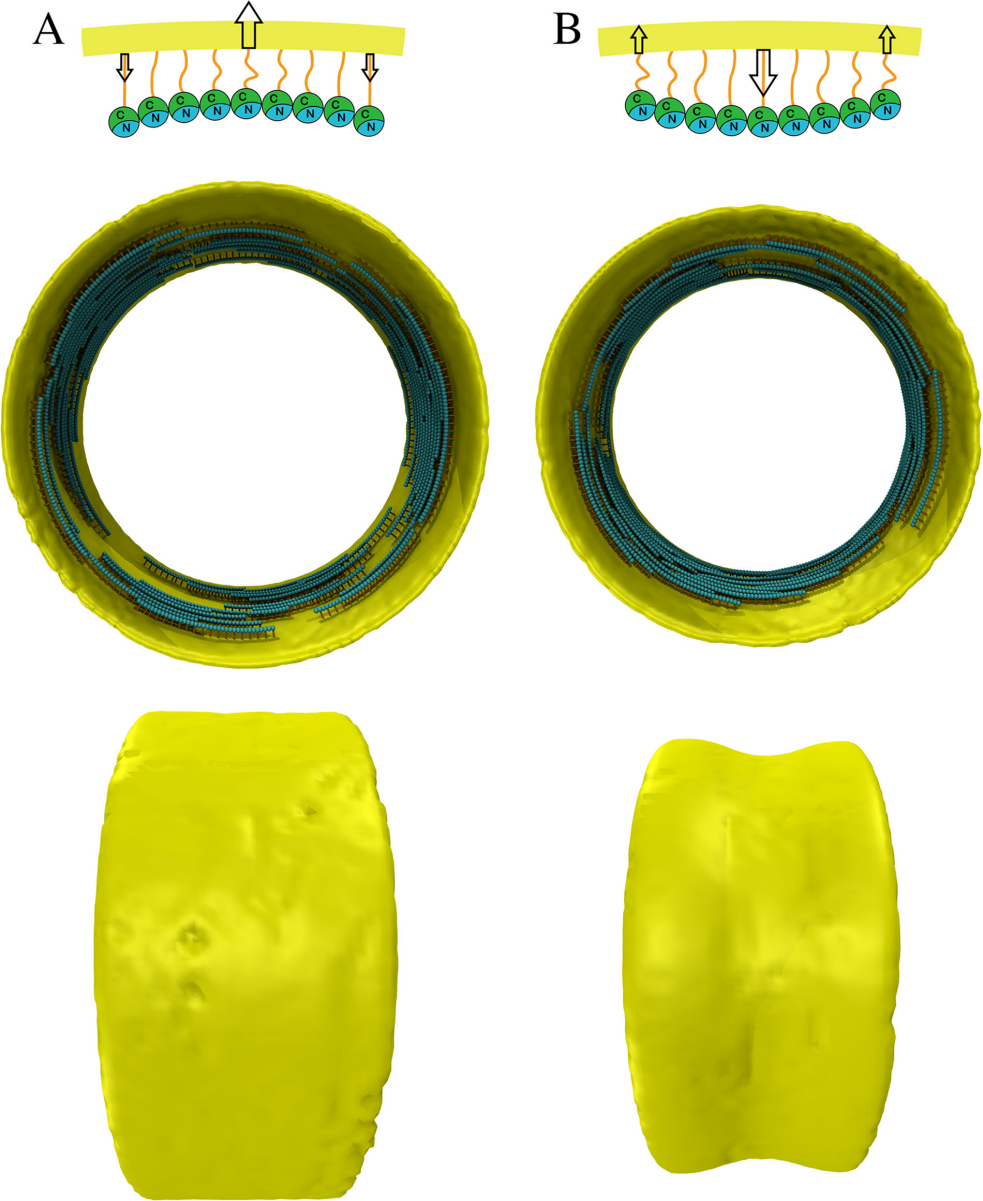
(A and B) Traditional bending (A) versus reverse bending (B). (Top) Schematic of how filament bending has traditionally been thought to occur versus bending in the opposite direction to the membrane (“reverse” bending). In both cases, the C termini face the membrane. Arrows indicate the directions of forces exerted on the membrane by the filament. (Middle) Simulation results after 100 s of systems in which the membrane initially had a diameter of 500 nm and the preferred curvature of the filament was 1/250 nm^−1^ (twice that of the initial membrane). Traditional bending did not result in constriction, but reverse bending did. (Bottom) Side views.

In the presence of the rigid linker, the “circumferentially constrained linker” condition was no longer motivated by the need to prevent filament rolling, but we found that it prevented filaments from becoming parallel to the long axis of the cell and treadmilling away from the midplane. With these rules in place, constriction resulted from the collective action of many short filaments that individually bent and exerted force on the membrane locally (Fig. 4A). This condition was consistent with the observation via cryo-ET by our lab and others (9, 10, 27) that most FtsZ filaments in dividing cells are short (∼100 nm).

As mentioned above, intuitively, the constrictive force depends on the difference in curvature between the membrane and the filament. Therefore, as the membrane becomes smaller, the constrictive force is reduced. When the resistance from the membrane bending stiffness and turgor pressure balances the constrictive force from the filaments, constriction should stop. Indeed, except for cases where the filaments were allowed to roll on the membrane, in our simulations, we never observed filaments that bent to their fully preferred curvature. In our initial simulations, FtsZ filaments were set to bend to a small radius, consistent with the observation of 24-nm minirings *in vitro* (28). However, in several other *in vitro* studies, FtsZ filaments formed large rings with diameters ranging from 150 to 1,000 nm (30–34, 49–51). We therefore ran additional simulations in which the preferred diameter of the curved filaments was only half (250 nm) that of the initial membrane (500 nm). As expected, these simulations did not result in membrane constriction (Fig. 4A), confirming that a substantial force is required for bending-driven constriction.

**(iv) Reverse bending.** While Söderström et al. have shown that FtsZ exits the ring before closure (52), it remains unclear exactly when. Using physical reasoning, Erickson et al. (8) pointed out that even minirings of 24 nm diameter (28) could only constrict the membrane to an ∼56-nm diameter, taking into account the FtsZ-membrane distance of ∼16 nm. This assumes that the filament bends in the same direction as the membrane. In this arrangement, a bent filament pushes the membrane outward at the center of the filament and pulls the membrane inward at the two ends (Fig. 4A). It has been thought that this is how FtsZ filaments bend, attached by their C-terminal face to the membrane via linkers and bending toward their N-terminal side. Li et al., however, found evidence that FtsZ filaments bend toward their C-terminal side, and so proposed models in which the C-terminal side faced the center of the cell (53). This would mean that the C-terminal linker would have to wrap around the filament to reach the membrane (Fig. S12). While this seemed to us unlikely, the paper caused us to realize that if the filament did bend toward its C-terminal side, and if the C-terminal side faced the membrane as originally envisioned, bending would pull the membrane inward at the center of the filament and push outward at the ends (Fig. 4B). In this “reverse bending” arrangement, even slightly bent filaments might be able to constrict the membrane. To explore the idea, we repeated simulations in which the preferred diameter of the curved filaments was only half (250 nm) that of the initial membrane (500 nm), but with the modification that the filaments were now modeled to bend in the opposite direction of the membrane. This modification changed the result; in this case, the membrane constricted even though the filament curvature was small (Fig. 4B). We note that this constrictive mechanism could persist until the diameter of the cell was just a few FtsZ monomers across. Again, constriction was the result of a collective action of many short treadmilling filaments, each bending and exerting force on the membrane locally.

**FIG 5:**
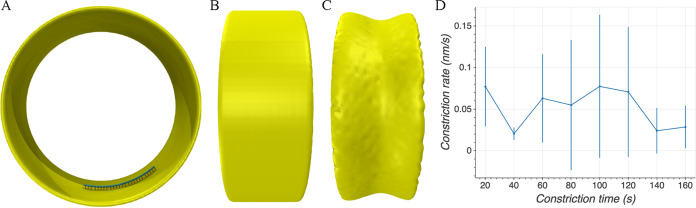
Single-filament constriction. (A) Axial view of the initial single-filament system. (B) Side view of the initial system. (C) Side view of the system after 100 s of simulation time showing that even a single FtsZ filament can constrict the membrane through reverse bending and treadmilling. (D) Cell wall constriction rate averaged over four simulations. Error bars indicate standard deviation.

**(v) Single-filament constriction.** A recent cryo-ET study from our lab showed that in several species, constriction initiates with a single FtsZ filament visible (27). Since a single filament can only exert force locally on the membrane, we speculated that the filament must connect to the cell wall. If so, as the filament bends, its end-to-end distance would decrease, and this could locally relax the pressurized cell wall (Fig. S13). This relaxation of the cell wall creates the condition that our previous simulations suggested is required to allow new cell wall of a smaller diameter to be incorporated (4). We therefore ran simulations of the reverse bending model in which there was only a single FtsZ filament with a preferred curvature of 1/32 nm^−1^ and again observed membrane constriction (Fig. 5).

## DISCUSSION

How bacterial cells generate a constrictive force for division without motor proteins remains a puzzle. To explore possible mechanisms, here, we designed the software ZCONSTRICT and performed simulations to test two popular conceptual models.

For the filament sliding model to work, we found that it requires the following conditions. First, there must be a long-range attractive force between the filaments. While we are not aware of any physical basis for such an attractive force in real cells, it is certainly possible that some connecting protein binds between filaments. Second, as sliding increases lateral contact and avidity, a mechanism such as depolymerization or treadmilling is needed to maintain a low number of lateral bonds to sustain sliding. Previous work by Lan et al. using Monte Carlo simulations predicted that sliding can occur since the lateral energy of the system decreases as the lateral contact increases (26). The authors evaulated only the energy of states where beads on one filament are in perfect register with those on the partner filament, and their evaluation is consistent with ours for these “in-register” states. However, similar to the evaluation by Erickson (54), our calculations showed that the energy barrier between adjacent in-register states (in other words, the cost to break the bonds and transition through an intermediate state where the beads are not in register) increases with the number of lateral bonds, eventually preventing sliding. Third, sliding can generate ring tension only if filaments overlap to form a complete ring. Cryo-ET imaging studies from our lab and others observed complete rings in mid- and late stages of constriction (10, 27), but in early stages in several species, we observed constriction without a complete ring (9, 27). Thus, some other mechanism must be at work at least initially.

In the filament bending model, it is proposed that GTP hydrolysis drives FtsZ filaments to switch from a straight to a bent conformation, constricting the membrane. For this model to work, we found that first, the filaments must be constrained to bend within the division plane (as predicted by Erickson et al. [8]). While we found in our simulations that a rigid linker could provide this constraint, we are not aware of any evidence for such a linker *in vivo*, and in fact, the known linkers have been shown to be flexible. Second, if the filaments bend in the same direction as the membrane, a large difference in curvature between the membrane and the filaments is needed for filament bending to overcome resistance from membrane stiffness and turgor pressure, and the final curvature is limited by the natural relaxed curvature of the filaments. If filaments bend in the opposite direction, however, constriction can proceed with even small filament curvatures and proceed nearly to fission. This might explain the observation that FtsZ filaments bend toward their C-terminal face, which is attached via linkers to the membrane (53). A more recent study, however, showed evidence that FtsZ bends toward its N-terminal face (55), so evidence for both same- and reverse-sense bending exists (53, 55).

Only the filament bending model can explain initial constriction with a single short filament (27), as seen in some cryo-ET reconstructions of dividing cells. While FtsZ filaments are highly dynamic, and it is possible that other filaments depolymerized just before the cells were frozen, the observation nevertheless strongly suggests that complete rings are not required. How could a single short filament drive constriction by bending? We showed previously that for constriction to proceed, turgor pressure must be overcome so that glycan strands in the cell wall relax, so new, shorter glycan strands can be added (4). This is possible, even for a single short filament, if the filament is attached to the cell wall, for instance through other proteins of the divisome (56).

As with any simulation of a conceptual model, our work has obvious limitations. First, we were only able to model a limited number of cellular components—the membrane, cell wall, FtsZ filaments, and linker proteins. Second, we assumed the cell wall does not actively push on the membrane but simply passively grows in after the membrane. While our previous work provides support for this assumption (4), it has not yet been validated experimentally. Third, due to the large scope of our simulations, we could explore only a finite parameter space for each conceptual model. While we believe the essential conclusions all stand to reason, the detailed particle trajectories are, of course, specific to the parameter set we tested. Finally, of course, it is possible that FtsZ generates a constrictive force by some other mechanism not modeled here. For example, FtsZ has recently been shown to treadmill around the cell circumference (14, 15). While treadmilling was speculated to be a mechanism to uniformly distribute new cell wall material around the division site, such dynamics have the potential to generate movement of other proteins, and perhaps those proteins somehow generate a constrictive force. The fact that purified FtsZ can constrict membranes alone (35–38) argues against this. The size mismatch between FtsA and FtsZ has also been speculated to cause filament bending (57). In this model, FtsA forms a filament parallel to and just outside the FtsZ filament (between FtsZ and the membrane). The difference in the FtsA monomer length (4.8 nm) and the FtsZ monomer length (4.4 nm) and the one-to-one connection between the two causes bending. Cryo-ET reconstructions of dividing cells do not support this model, however, as only a single layer of filaments is seen in cross sections perpendicular to the long axis of the cell, ∼16 nm from the membrane. A recent study showed long filaments could constrict the liposomes into a helical shape (58), but we have never seen FtsZ filaments that long and wrapping around the cell in our cryo-ET reconstructions of dividing cells. Other force generation mechanisms that do not even involve FtsZ are also possible, for instance, excess membrane synthesis, which has recently been proposed (58). Thus, our purpose was not to prove how or even if FtsZ generates constrictive force but, rather, to explore the conditions required for the two most popular models to function as envisioned. We hope this will sharpen future experiments that ultimately reveal which mechanisms are in play in real cells.

## MATERIALS AND METHODS

In this section, we describe the design of the software ZCONSTRICT and implementation of assumptions of our simulations.

### Membrane.

Similar to our previous work (5), we modeled the membrane as a sheet of beads originally forming a cylinder (Fig. 1). In all of our simulations, the original membrane cylinder was 160 nm wide and 250 nm in radius. The bead size was set at *d*_mb_ = 8 nm such that if the distance *d* between two beads was smaller than *d*_mb_, a force *F*_push_ = *k*_pair_(*d*_mb_ – *d*)^2^ was applied on each bead to push them apart, where *k*_pair_ = 1 pN/nm^2^ was the pairwise force constant. To preserve membrane integrity, a pairwise attraction was implemented between neighboring beads such that if the distance *d* between two beads was larger than *d*_pair_ = 16 nm, a force *F*_pull_ = *k*_pair_(*d* – *d*_pair_)^2^ was applied on each bead to pull them together. To implement membrane fluidity, the pair list was recalculated every 10^4^ time steps. As different pairs were formed based on the updated positions of the beads, the beads were allowed to move along the membrane. In our previous membrane model (5), a pair was removed from the pair list if it was crossed (looking outward from the center) by a shorter pair. In the current model, to avoid a large change in force on the system as the pair list was recalculated, one degree of crossing was allowed, meaning that a pair was removed from the pair list only if it was crossed by more than one shorter pair.

To check how sensitive our simulations were to changing the value of *k*_pair_, we also ran simulations of the filament sliding models with this parameter reduced 10-fold (*k*_pair_ = 0.1 pN/nm^2^) and increased 10-fold (*k*_pair_ = 10 pN/nm^2^). However, we did not see any effect in the membrane morphology but only a small effect on the constriction rate (see Fig. S14 in the supplemental material).

To implement membrane bending stiffness, if four beads were part of five pairs (Fig. S15), they were constrained to the same plane. If the two diagonals were separated at a distance *d*, a force *F*_mb_ = *k*_mb_*d* was exerted on the beads to pull the two diagonals together, where *k*_mb_ was a force constant. To derive *k*_mb_, we first built a coarse-grained model of the membrane as a cylindrical sheet of beads with length *L* = 160 nm and radius *R* = 250 nm. We calculated the bending energy of this membrane model $E_{\mathrm{mb}\_\mathrm{sim}}=2\sum k_{\mathrm{mb}}d^{2}/2$ (the factor of 2 comes from the fact that *F*_mb_ was exerted on four beads). Next, using an experimentally reported membrane bending stiffness *k*_*m_*exp_ ∼ 2 ⋅ 10^−19^ J (59), we calculated the bending energy of a cylindrical membrane as *E*_mb_exp_ = *k*_*m_*exp_π*L/R*. Comparing the two energy calculations, we derived *k*_mb_ to be 8 pN/nm.

Similar to our previous membrane model (5), to prevent boundary artifacts, we implemented a periodic boundary condition such that images of the beads on one longitudinal edge were translated to interact with the beads on the other edge. The translational distance was the same as the membrane width.

### Filament sliding model.

In this model, the FtsZ filament was modeled as a chain of beads, each bead representing one FtsZ monomer. The adjacent beads were connected by springs of a relaxed length of *l_z_* = 4.4 nm (28) and a spring constant chosen to be *k_z_* = 0.5 nN/nm, which was sufficiently large to prevent significant stretching or compression of the filament. If the filament bent an angle θ at a bead, an energy *E_θ_* = *k_θ_*(θ – θ_0_)^2^/2 was added to the system, where θ_0_ = 0° was the relaxed angle. We derived the bending stiffness constant as *k_θ_* = *k_B_TL_p_*/*l_z_* = 3.8 ⋅ 10^−18^ J, where *k_B_* is the Boltzmann constant, *T* = 295 K is room temperature, and *L_p_* = 4 μm is the filament persistence length (60). Note that much smaller values of FtsZ persistence length ∼100 nm have also been reported (18, 33).

We implemented a Lennard-Jones potential as a long-range interaction between beads on different filaments (note that interaction of beads on the same filament was excluded). Specifically, a force between two beads at a distance *d* was calculated as

(1) $$F_{1}=\frac{24\varepsilon }{d}\left[2{\left(\frac{\rho }{d}\right)}^{12}-{\left(\frac{\rho }{d}\right)}^{6}\right]$$

where the distance at which the potential is zero was set to be *ρ* = 6.5 nm, about the distance between paired filaments observed via cryo-ET of dividing cells (10). Varying the depth of the potential *ε* in the range from 10^−19^ to 10^−18^ J with a step size of 10^−19^ J, we found that the lower bound was too weak to result in filament sliding, while values larger than 5 ⋅ 10^−19^ J resulted in filaments twisting around each other. We therefore chose *ε* = 5 ⋅ 10^−19^ J, the largest value that induced filament sliding without causing filament twisting.

To implement physical separators to keep different rings apart, if the distance *d* between two FtsZ beads on two separate rings became smaller than *d_s_* = 20 nm, a repulsive force *F_s_* = *k_s_*(*d* – *d_s_*) was applied on the beads to push them apart. The force constant *k_s_* was chosen to be 50 pN/nm, sufficiently large to efficiently separate rings.

To implement filament depolymerization and treadmilling, the FtsZ filament was modeled to be polar with one end tracked to be “plus” and the other “minus.” Depolymerization was modeled by removing a bead at the minus end with a rate varying from 1 to 10 beads/s. Treadmilling was modeled by adding a bead to the plus end and removing a bead from the minus end at the same rate, which was varied from 1 to 14 beads/s.

### Filament bending model.

We assumed that the filament bends in the same plane without twisting. To implement this assumption, we modeled the filament as a chain of cubes, with each cube representing one FtsZ monomer (Fig. S9). For convenience, the beads on each cube were denoted 1, 2, 3, 4, 5, 6, 7, and 8. Note that the beads on the interface of two adjacent cubes were shared, meaning beads 5, 6, 7, and 8 of one cube were the same as beads 1, 2, 3, and 4 of the other. The C-terminal face contained beads 1, 2, 6, and 5, and the N-terminal face contained 4, 3, 7, and 8. To maintain the width of the filament, the beads on the two cross-sectional faces (perpendicular to the long axis; one containing 1, 2, 3, and 4, and the other containing 5, 6, 7, and 8) were connected by springs of relaxed length *l_z_* = 4.4 nm and spring constant *k_z_* = 0.5 nN/nm (Fig. S9A). Note that calculation of the spring force on the interface of two adjacent cubes was done only once for each spring. To maintain the length of the filament, the two cross-sectional faces were connected by two springs of relaxed length *l_z_* and spring constant *k_z_*. One spring connected edge 1-4 to edge 5-8, and the other connected edge 2-3 to edge 6-7 (Fig. S9B).

To model filament bending, the beads on the C-terminal face were connected by two springs of relaxed length *l_C_* and spring constant *k_b_*, one spring connecting bead 1 to bead 5 and the other connecting 2 to 6 (Fig. S9B). Likewise, on the N-terminal face, one spring of relaxed length *l_N_* and spring constant *k_b_* connected bead 3 to bead 7, and another (identical) spring connected 4 to 8. When the filament was in a straight conformation (supposedly GTP-bound), both *l_C_* and *l_N_* were set to be *l_0_*, which was the distance between the two centers of the two cross-sectional faces (Fig. S9B). Note that *l_0_* was not constrained to be constant but varied with the stretching/compression of the filament. When the filament was in a bent conformation (having already hydrolyzed GTP) with a preferred angle *θ_b_* between adjacent monomers, *l_C_* was set to be *l_0_* + Δ*l_0_* and *l_N_* was set to be *l_0_* – Δ*l_0_*, where $\Delta l_{0}=l_{z}\text{sin}(\frac{\theta_{b}}{2})$ (Fig. S9C). In the case that reverse bending was assumed, *l_C_* was set to be *l_0_* – Δ*l_0_* and *l_N_* was set to be *l_0_* + Δ*l_0_*. In our simulations, we varied *θ_b_* between 1° and 20°. Considering the distance between adjacent subunits *l_z_* = 4.4 nm, bent filaments that form circles with diameters of 250 and 50 nm correspond to *θ_b_* = 2° and 10°, respectively. The spring constant *k_b_* was calculated by setting the energy stored in four springs as their length was deformed an amount Δ*l_0_* to be the bending energy of the filament with bending stiffness *k_θ_* (the same constant as in the filament sliding model) as follows:

(2) $$4(\frac{1}{2}k_{b}\Delta {l_{0}}^{2})=\frac{1}{2}k_{\theta }{\theta_{b}}^{2}$$

Note that *θ_b_* was converted to radians when the bending energy was calculated.

To prevent filament twisting, the two diagonals of each face were constrained to the same length such that if their lengths differed by an amount Δ*l*, a force *F*_diag_ = *k_z_*Δ*l* was applied on the four beads to restore the lengths to the same value (Fig. S9D).

### Filament-membrane connection.

Each filament was connected to the membrane via linkers (Fig. 1). Each linker was composed of two identical springs of a spring constant of *k_lk_* = 20 pN/nm and a relaxed length of *l_lk_* = 8 nm, making the distance from the filament to the membrane 16 nm as reported experimentally (9, 10). One spring was connected to an FtsZ monomer, and the other, to a membrane bead, and they were joined by a bead representing FtsA or ZipA. In the case of the filament bending model, because each FtsZ monomer was represented as a cube, the associated linker was linked to the cube’s center.

### Circumferentially constrained linkers.

To constrain the linkers connected to the same filament to the circumferential direction, if two membrane beads that were connected to two adjacent linkers were separated at a distance *d* along the axis perpendicular to the circumferential direction (Fig. S16), a restoring force, *f_c_* = –*k_c_d*, where *k_c_* = 20 pN/nm was a force constant, was applied to align them back to the circumferential direction.

### Rigid linkers.

To make the linker rigid, the original linker model as two springs of a relaxed length of 8 nm and a spring constant *k_lk_* = 20 pN/nm joined at a central bead was replaced with a single spring of 16 nm long and the same spring constant *k_lk_*. To constrain the linker to the radial direction of the membrane, if the two ends were separated at a distance *d* along the axis that was perpendicular to the preferred (radial) direction (Fig. S16), a restoring force, *f_r_* = –*k_r_d*, where *k_r_* = 3 pN/nm was a force constant, was applied on the ends to align them back to the radial direction. To constrain filament rolling, the springs connecting the C- and N-terminal faces of the FtsZ-monomer cubes, specifically those that connect bead 1-4, 2-3, 5-8, and 6-7 (Fig. S9), were constrained parallel to the division plane (the plane perpendicular to the cell long axis). If a constrained spring deviated from the division plane, a restoring force of magnitude *F*_dp_ = *k*_dp_*d*, where *k*_dp_ = 20 pN/nm was a force constant and *d* was the projection of the spring length on the cell long axis, was applied on each of the two end beads of the spring to align it to the division plane (Fig. S17).

### Cell wall.

Previously, we modeled the cell wall of Gram-negative bacteria as hoops of glycan strands connected by peptide cross-links in which each strand was modeled as a chain of beads and each hoop was composed of several strands (3, 4). In the current work, we did not focus on the dynamics of the cell wall. We therefore simplified our cell wall model to reduce the computational cost. Specifically, the cell wall was modeled as a grid of beads composed of 11 hoops of radius *r_g_* = 265 nm, separated at a distance of 16 nm and originally forming a cylinder 160 nm wide (Fig. 1). There were 104 beads per hoop separated at a distance of 16 nm from each other and 15 nm from the membrane. The beads on the grid were connected to each other by two types of springs. The glycan springs with spring constant *k_g_* = 100 pN/nm and dynamic relaxed length *l_g_* ran along the circumference. The peptide springs of spring constant *k_p_* = 10 pN/nm and relaxed length *l_p_* = 16 nm ran along the cell’s long axis. Initially, *l_g_* was chosen such that turgor pressure stretched the glycan spring to a length of *l_ext_* = 16 nm. As the turgor pressure was chosen to be *P_tg_* = 1 atm, the original *l_g_* was calculated to be

(3) $$l_{g}=l_{\mathrm{ext}}-\frac{P_{\mathrm{tg}}r_{g}}{k_{g}l_{p}}$$

To prevent boundary artifacts, the two outermost hoops on the two edges of the wall cylinder were treated as translational images of each other with the translational distance the same as the edge-to-edge distance. These two hoops locate at the same radial and angular coordinates and were subjected to the same force.

### Turgor pressure.

To calculate the force from turgor pressure on the cell wall beads, we first calculated the volume *V* enclosed by the cell wall. To do this, each tetragon was divided into two triangles by one diagonal. Each triangle together with the center point of the cell wall formed a tetrahedron. The total volume *V* was the sum of *V_i_*, which was the volume of tetrahedron *i*. The force on each bead *j* was then calculated as $F_{j}=P_{\mathrm{tg}}\sum \nabla_{j}V_{i}$. As mentioned above, turgor pressure was chosen to be *P*_tg_ = 1 atm.

### Cell wall-membrane connection.

We assumed that the cell wall bears all the force from turgor pressure and that this force is transmitted through the membrane and a buffer layer of periplasmic proteins between the membrane and the wall. Similar to our previous model of the membrane (5), we modeled the membrane as squeezable, allowing the distance between the membrane and the cell wall to vary around the equilibrium distance of *d_w–m_* = 15 nm. As the distance from a membrane bead to the cell wall deviated Δ*d* from *d_w–m_*, a force *F_w_* = *k_w_*Δ*d*^2^ (representing the net force from the cell wall and turgor pressure) was applied on the membrane bead to restore it to the equilibrium distance. The force constant was chosen to be *k_w_* = 1.6 pN/nm^2^.

### Cell wall growth.

To model cell wall growth, as the membrane was pulled down, the relaxed lengths of two glycan springs connected to the cell wall bead that was closest to the membrane bead were shortened so the wall could move inward to fill the gap. Specifically, every 10^4^ time steps, we calculated the simulated duration Δ*t* and the maximal allowed inward displacement of the cell wall *ν_w_*Δ*t*, where *ν_w_* = 100 nm/s was the limit of the cell wall inward growth rate. If the distance between a membrane bead and the local cell wall surface became larger than *d_w–m_* = Δ*d_m_*, where Δ*d_m_* = 0.5 nm, the inward displacement of the local cell wall bead Δ*d_m_* was chosen as the smaller of either 0.05 nm or *ν_w_*Δ*t*. Note that this rule resulted in an average inward growth rate of the cell wall ν_radial_ = 14 nm/s. The relaxed lengths of the two glycan springs connected to the local cell wall bead were then shortened an amount πΔ*d_w_*/*N_b_*, where *N_b_* = 104 was the number of wall beads per hoop.

### Calculation of the constriction rate.

The constriction rate was defined as the average reduction rate of the cell wall radius. Specifically, the constriction rate between time *t_0_* and time *t* was calculated as

(4) $$\nu_{\mathrm{radial}}=-\frac{\sum r_{i}(t)-\sum r_{i}(t_{0})}{N_{w}\Delta t}$$

where *r_i_* was the radius of bead *i* and *N_w_* was the number of beads on the cell wall, Δ*t* = *t – t*_0_.

### Diffusion.

As in our previous simulation work (3–5), we modeled the thermal motion of the system by implementing random forces on the beads. We used the Box-Muller transformation to generate a set of Gaussian-distributed random numbers. Specifically, two random numbers of a Gaussian distribution were generated using two random numbers from a uniform 0 – 1 distribution, *u*_1_ and *u*_2_, as follows:

(5) $$r_{1}=\text{cos}(2\pi u_{2})\sqrt{-2\ln\left(u_{1}\right)}$$

(6) $$r_{2}=\text{sin}\left(2\pi u_{2}\right)\sqrt{-2\ln\left(u_{1}\right)}$$

To reduce the computational cost, we did not integrate the Gaussian distribution with a time step to generate random forces. Instead, a force was simply calculated as the product of a random number of the Gaussian distribution with a force constant *k_r_* varying from 1 to 10 pN.

### Volume exclusion.

We implemented a volume exclusion effect between FtsZ beads and membrane beads and between beads on different FtsZ filaments. If the distance *d* from an FtsZ bead to a membrane bead was smaller than *d_mb_* = 8 nm, a force *F* = *k_m–z_*(*d_mb_* – *d*), where *k_m–z_* = 200 pN/nm was a force constant, was applied on the beads to push them apart. In the filament sliding model, the Lennard-Jones potential provided a volume exclusion effect. In the filament bending model, if the distance *d* between the centers of two cubes (of two different filaments) was smaller than *d_zz_* = 6.5 nm, a force *F* = *k_zz_*(*d_zz_* – *d*), where *k_zz_* = 1 nN/nm was a force constant, was exerted on each bead of the two cubes to push them apart.

### System dynamics.

As in our previous simulations (3–5), we used the Langevin equation to calculate the system dynamics as follows:

(7) $$M\frac{d^{2}X}{dt^{2}}=-\nabla U\left(X\right)-\gamma \frac{\text{d}X}{\mathrm{dt}}+R(t)$$

where *M* represents the mass of the system, *X* is the coordinates, *U* is the interaction potential, *γ* is the damping constant, and *R* is the random force (see “Diffusion” above). To speed up the simulations, we used a large damping constant *γ* = 10^–7^ Ns/m.

We assumed that at a low Reynolds number, the inertia of the bead was negligible, and thus *M* = 0, the displacement in each time step was calculated as

(8) $$\mathrm{dX}=\frac{1}{\gamma }\left[-\nabla U\left(X\right)+R\right]dt=\frac{1}{\gamma }F\mathrm{dt}$$

which was linearly proportional to the total force *F*. To prevent the system from becoming unstable, the maximal displacement of any bead in one time step was constrained to be *d_max_* = 0.01 nm. The displacement of bead *i* at a time step was calculated as

(9) $$d_{i}=d_{\max}\frac{F_{i}}{F_{\max}}$$

where *F_i_* and *F_max_* were the force on bead *i* and the maximal force on the beads, respectively. The time step could then be calculated as

(10) $$\mathrm{dt}=\gamma \frac{d_{\max}}{F_{\max}}$$

which varied due to variation of *F*_max_$F_{\max}$. Each simulation was run for 1 billion to 10 billion steps and the average time step was ∼6 ns for the filament sliding model and ∼50 ns for the filament bending model.

The software ZCONSTRICT was written using Fortran (the source codes can be downloaded at https://github.com/nguyenthlam/Zconstrict). The trajectories of the system dynamics were visualized using VMD (Visual Molecular Dynamics) software (61).

## Supplementary Material

Supplemental file 1
