# Supplementary material for: Simulations of Proposed Mechanisms of FtsZ-Driven Cell Constriction
Source: J Bacteriol. 2021 Jan 11;203(3):e00576-20. doi: 10.1128/JB.00576-20 (PMC7811198; doi:10.1128/JB.00576-20)
Supplement: Supplemental file 1 [file supp_data_source_00576-20.pdf]

Supplementary Information

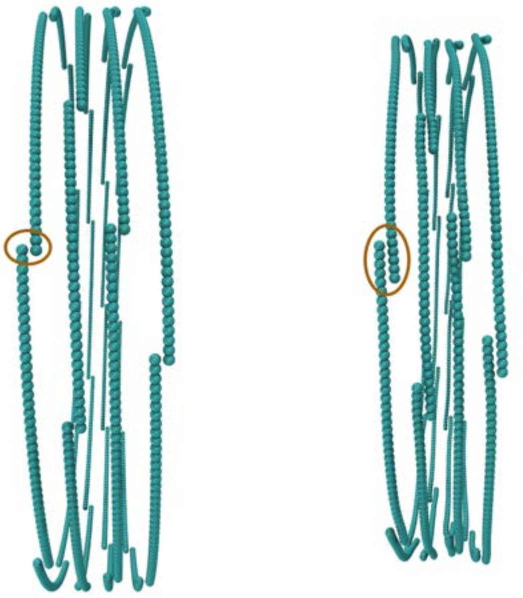

Figure S1: Filament sliding model with separated rings. Filaments in each ring were initiated with one bead overlapping (left). Filament sliding occurred but quickly stopped as overlapping reached ~5–6 beads (right). Ellipses indicate examples of overlap.

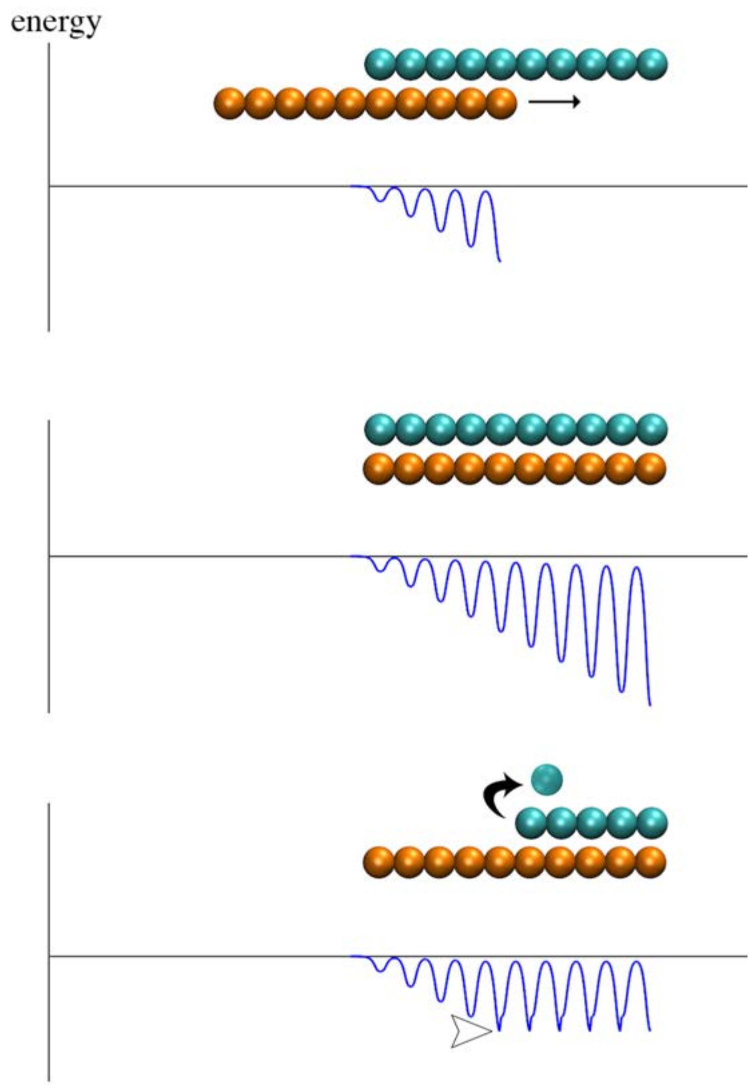

Figure S2: Lateral interaction energy of overlapping FtsZ filaments. (Top) As the orange filament slides (indicated by the arrow) with respect to the stationary cyan filament, their interaction energy oscillates with the minimum changing much faster than the maximum, resulting in an increasing energy barrier. (Middle) The energy barrier continues to increase with further filament overlap. (Bottom) If the stationary filament starts to depolymerize (arrow), the energy minimum remains constant (arrowhead), therefore maintaining a low energy barrier as filament sliding continues.

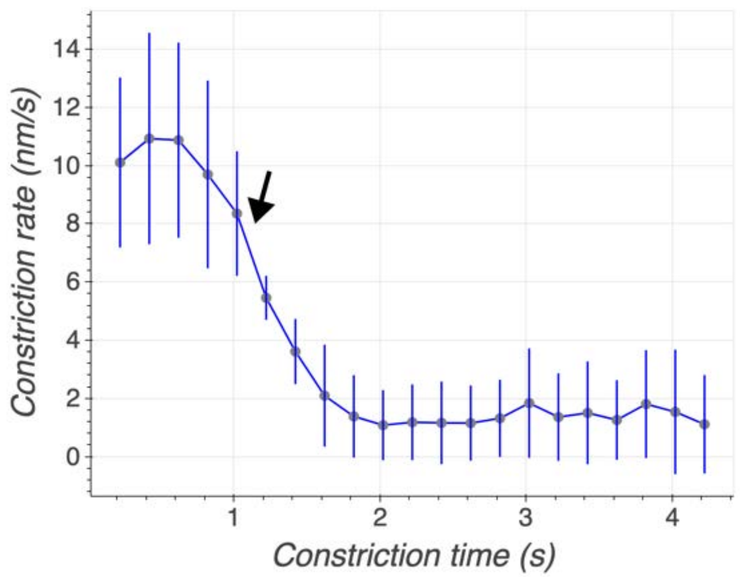

Figure S3: The constriction rate of the filament sliding model at a depolymerization rate of 5 beads/s. In these simulations, the maximum inward growth rate of the cell wall was set to be 100 nm/s. The arrow indicates loss of the ring integrity. Average was calculated over four simulations. Error bars indicate standard deviation.

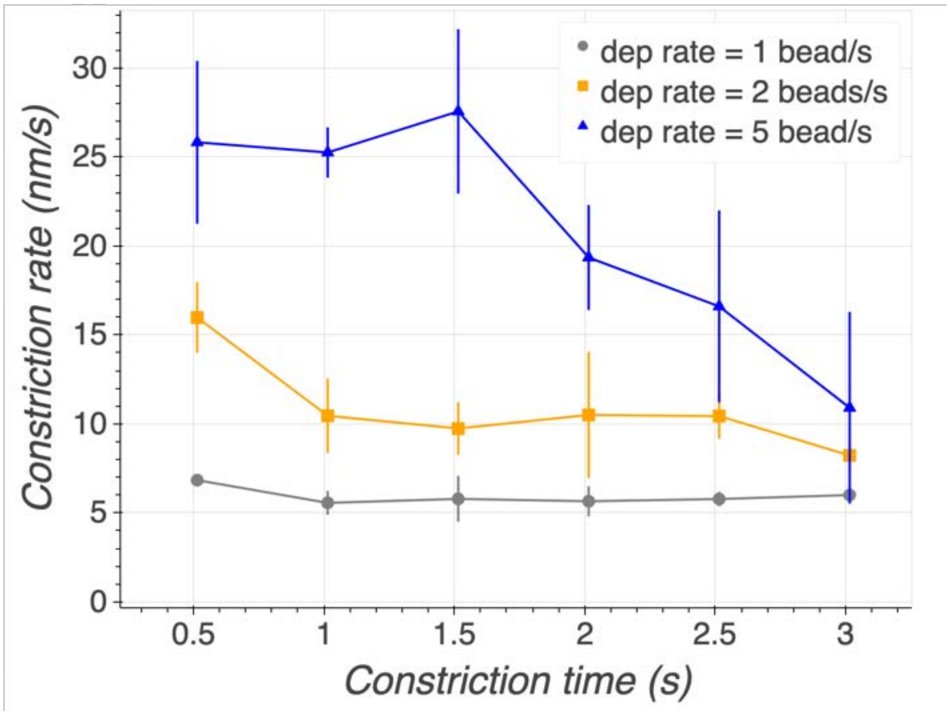

Figure S4: The constriction rate of the filament sliding model vs the depolymerization rate. In these simulations, the maximum inward growth rate of the cell wall was set to be 1000 nm/s. The filament depolymerization rate was therefore the only limiting factor of the constriction rate. Average was calculated over four simulations. Error bars indicate standard deviation.

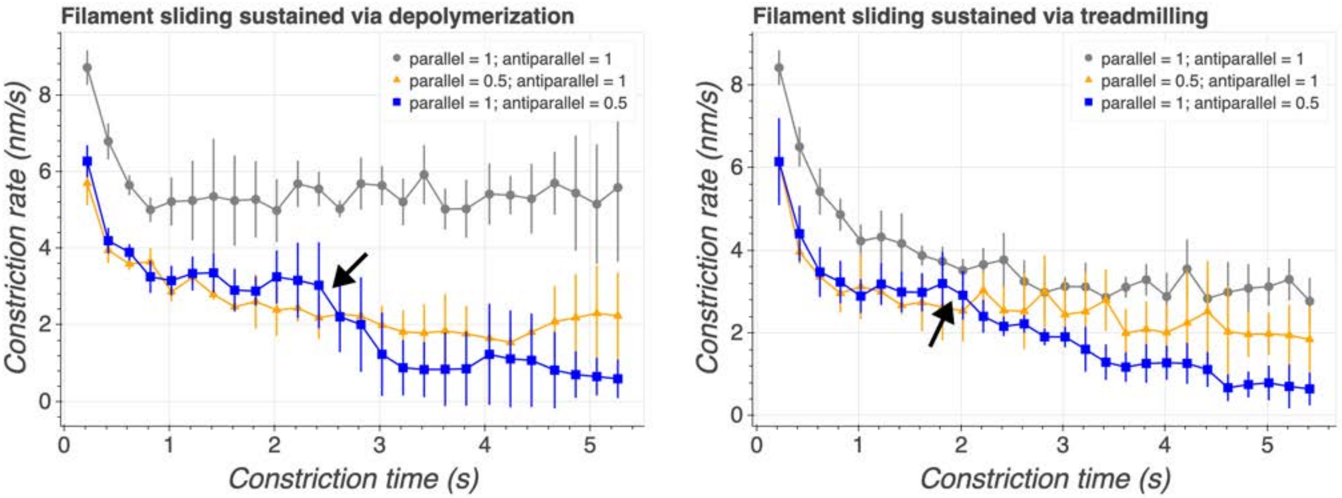

Figure S5: Comparison of effect of parallel interactions and that of antiparallel interactions. Time-series of the constriction rate of simulations in which filament depolymerization was implemented (Left) and treadmilling was implemented instead (Right). The constriction rate decreased if either the depth of Lennard-Jones potential ε for interactions between parallel filaments was reduced two folds from $5{\cdot}10^{-19}$ J to $2.5{\cdot}10^{-19}$ J (parallel = 0.5) or ε for interactions between antiparallel filaments was reduced two folds (antiparallel = 0.5). The latter led to a sudden reduction in the constriction rate corresponding to loss of the ring integrity (arrows). Legend: 1 indicates the potential depth $\varepsilon = 5{\cdot}10^{-19}$ J; 0.5 indicates $\varepsilon = 2.5{\cdot}10^{-19}$ J.

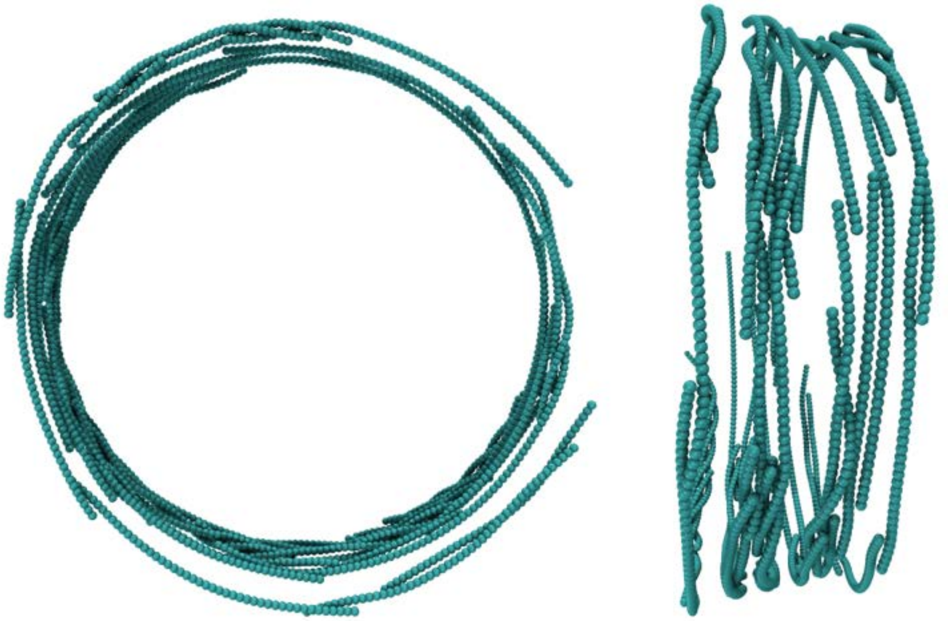

Figure S6: Implementation of treadmilling in the filament sliding model. An axial view (left) and a side view (right) of the constricted rings show that several filaments of the same ring overlapped to form bundles.

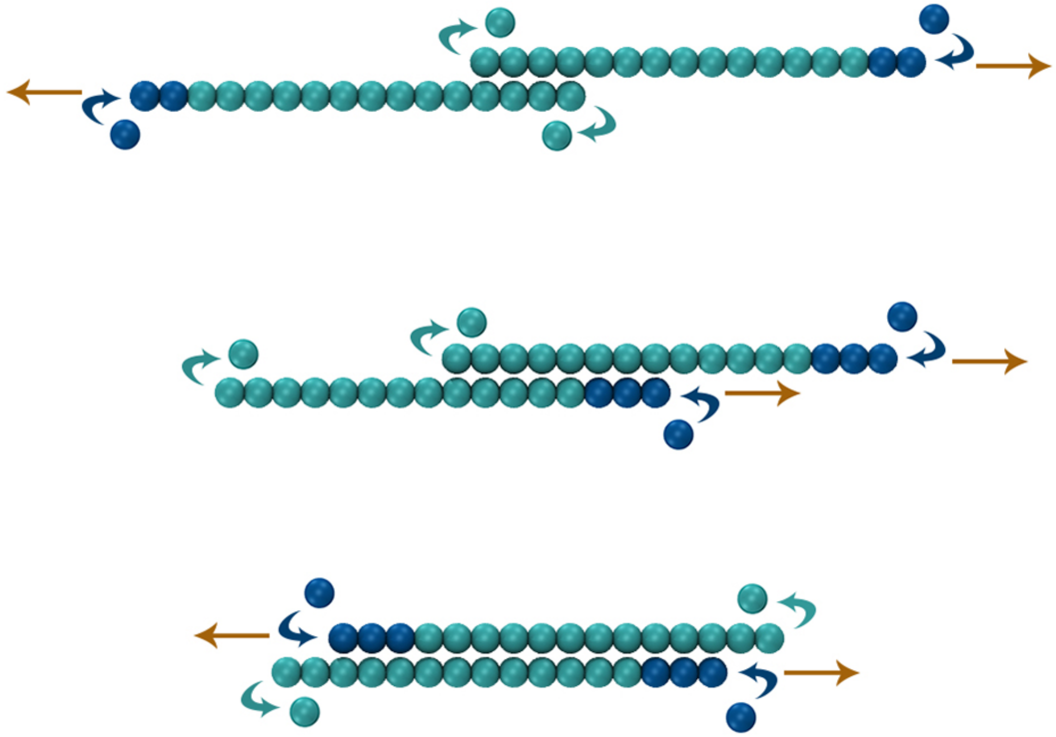

Figure S7: Three scenarios of filament treadmilling. Orange arrows indicate treadmilling direction. Dark blue arrows indicate the growing end of the filament. Cyan arrows indicate the shortening end of the filament. (Top) If two filaments treadmill away from each other, the number of their lateral bonds is reduced, allowing further filament sliding (similar to the effect of filament depolymerization). (Middle) If two filaments treadmill in the same direction, their overlap remains constant, causing no effect on filament sliding. (Bottom) If two filaments treadmill toward each other, their overlap increases, further increasing avidity.

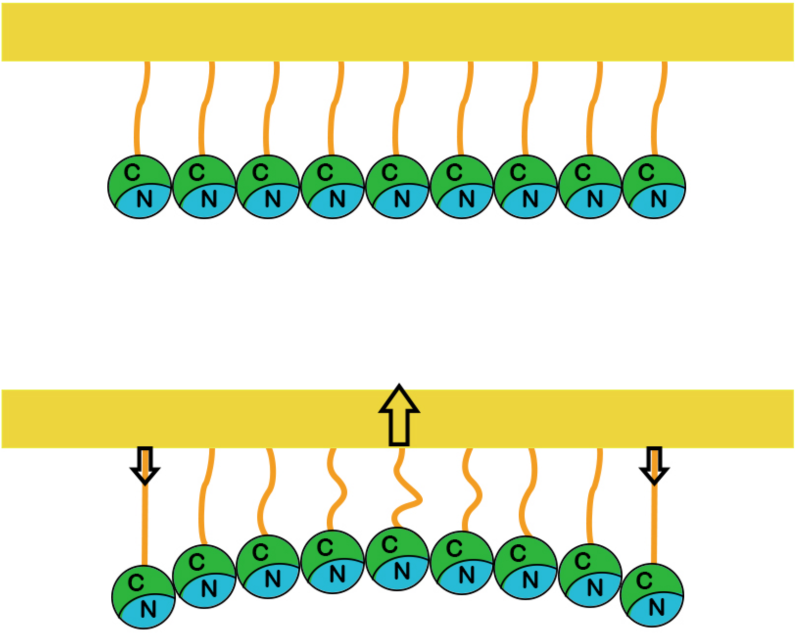

Figure S8: Schematic of the filament bending model. FtsZ is connected to the membrane at the C-terminus via flexible linkers. As the filament switches from straight (top) to bent (bottom) conformation, it exerts forces on the membrane. Arrows indicate the force directions.

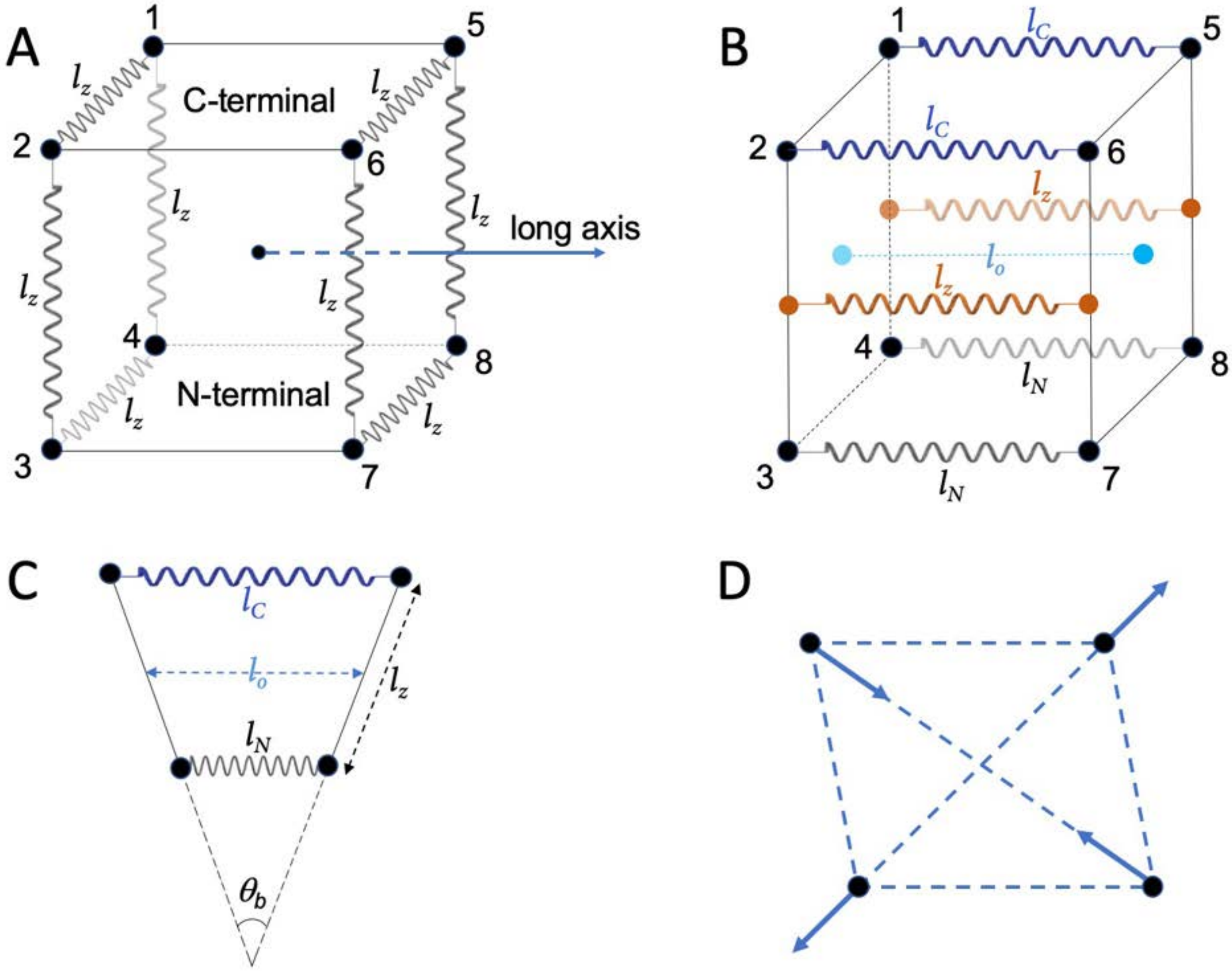

Figure S9: Cube model of the FtsZ filament. Each monomer is represented as a cube. Filament would be horizontal along the long axis. (A) The beads on each of the two cross-sectional faces are connected by identical spring of relaxed length l_z = 4.4 nm. (B) The two cross-sectional faces are connected by two springs (orange) of relaxed length l_z. One spring connects the center of edge 1-4 to the center of edge 5-8. The other spring connects the center of edge 2-3 to the center of edge 6-7. The centers of these four edges are indicated by the beads in orange. As the filament is in a straight conformation, the corners are connected by four springs of the same relaxed length, such that both l_C (blue) and l_N (black) are equal to l_0, which is the distance between the two centers of the two cross-sectional faces (cyan). (C) To model a bent conformation, l_C and l_N are increased and decreased, respectively. (D) Schematic of forces (arrows) exerted on the beads to restore a distorted face to a square shape.

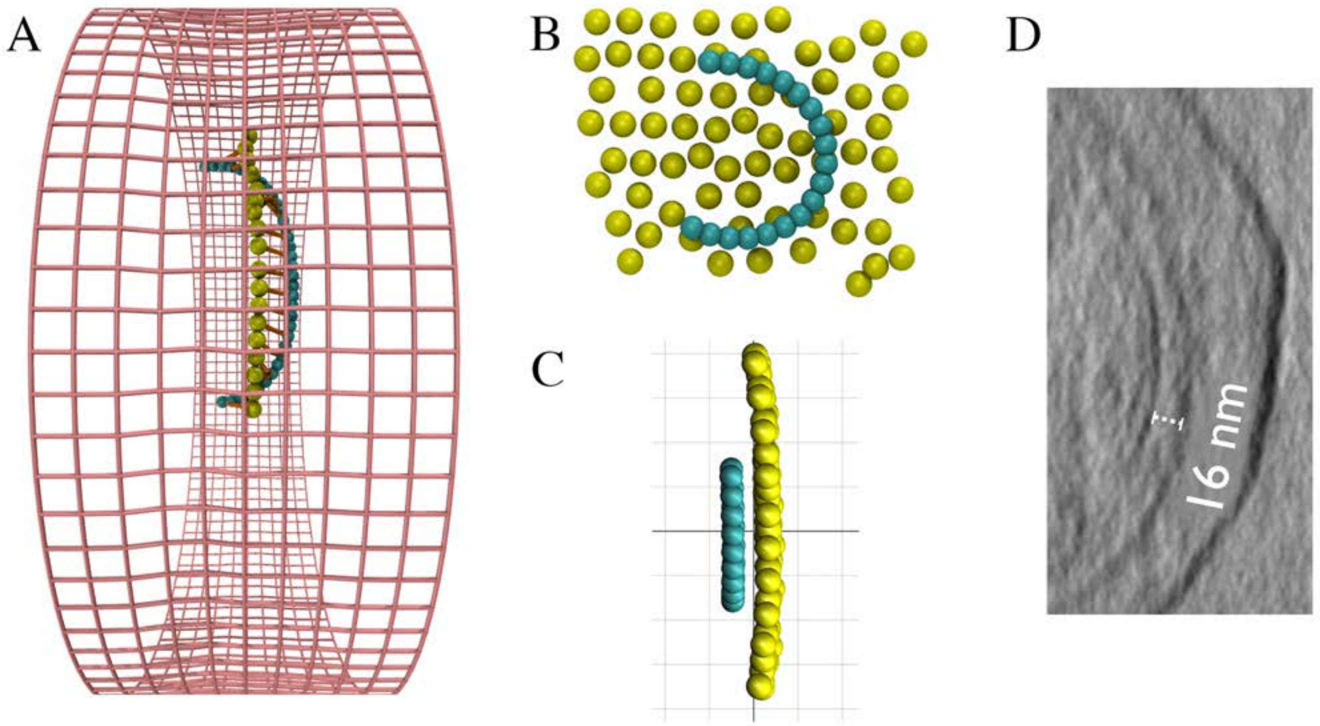

Figure S10: Adding a circumferential constraint on the linkers. (A) A filament and its connected membrane beads are visualized together with the cell wall to show that even when membrane beads were aligned to the circumferential direction, the flexibility of the filament and the linkers allowed filament rolling. (B) Front view and (C) side view of a filament with its neighboring membrane beads showing how the filament was pulled close (~8 nm) to the membrane. The grid size in (C) is 10 nm. (D) Adapted from Szwedziak et al. 2014, Figure 1C. A slice through an electron cryotomogram of a dividing cell. The distance from FtsZ to the membrane was measured to be 16 nm, consistent with Li et al. 2007.

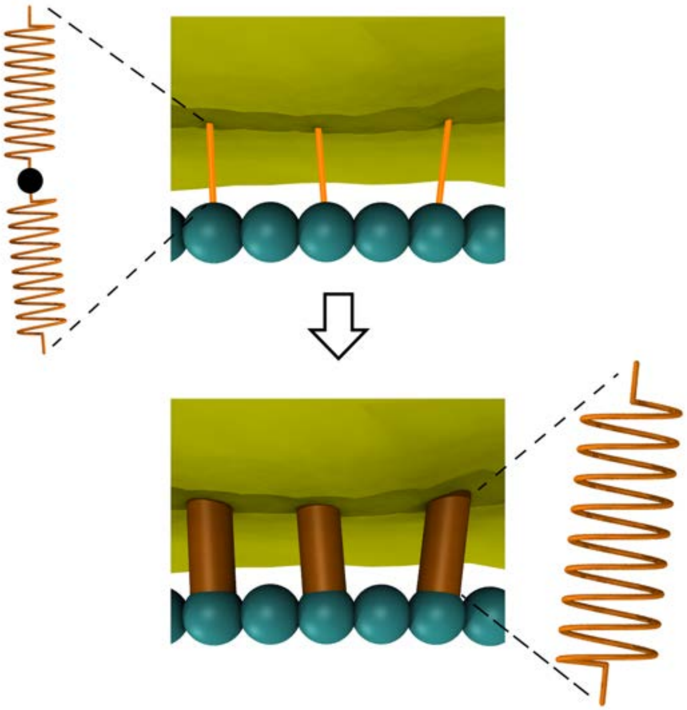

Figure S11: Schematic showing replacement of the flexible linker (that is composed of two springs and therefore can bend freely at the connecting bead) with a rigid linker to maintain the FtsZ-membrane distance and prevent filament rolling.

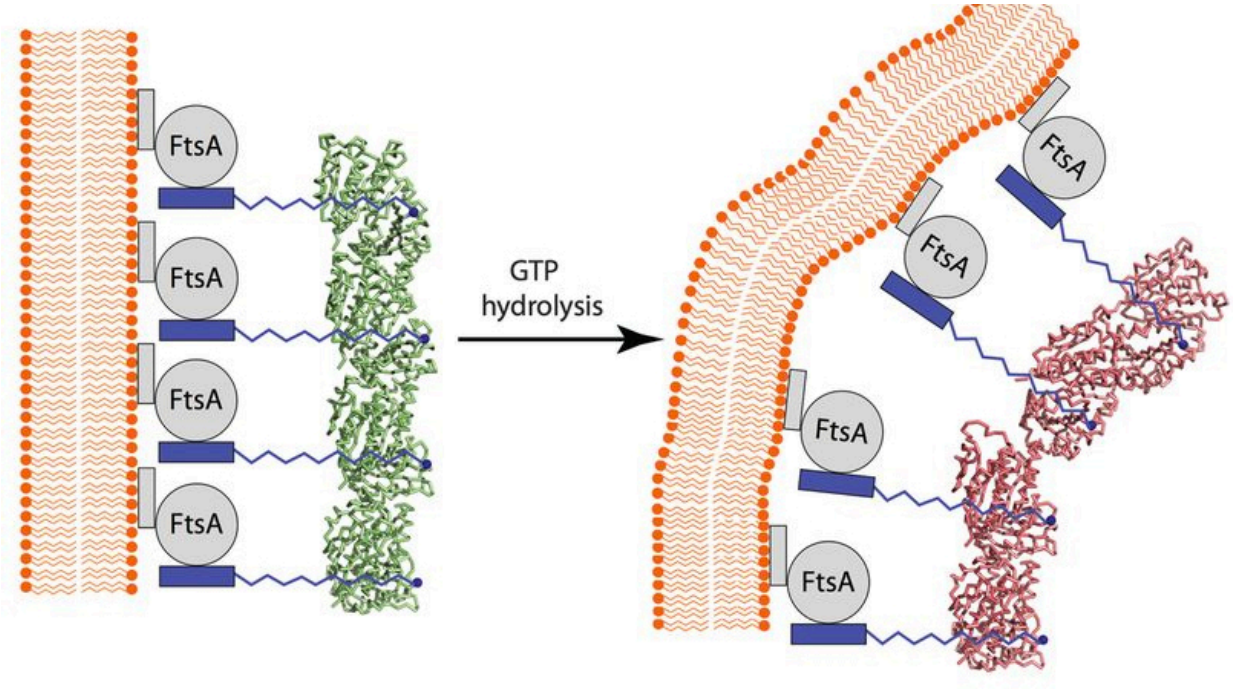

Figure S12: Adapted from Li et al. 2013, Figure 3A, in which the authors depicted a straight FtsZ filament (in light green) which upon GTP hydrolysis switched to a bent conformation (pink). The authors show evidence that the C-terminus of FtsZ is on the inner curvature of the bent filament. Assuming the filament bends in the same direction as the membrane, the authors interpreted this to mean that the flexible linker (blue) that connects FtsZ to FtsA must wrap around the filament.

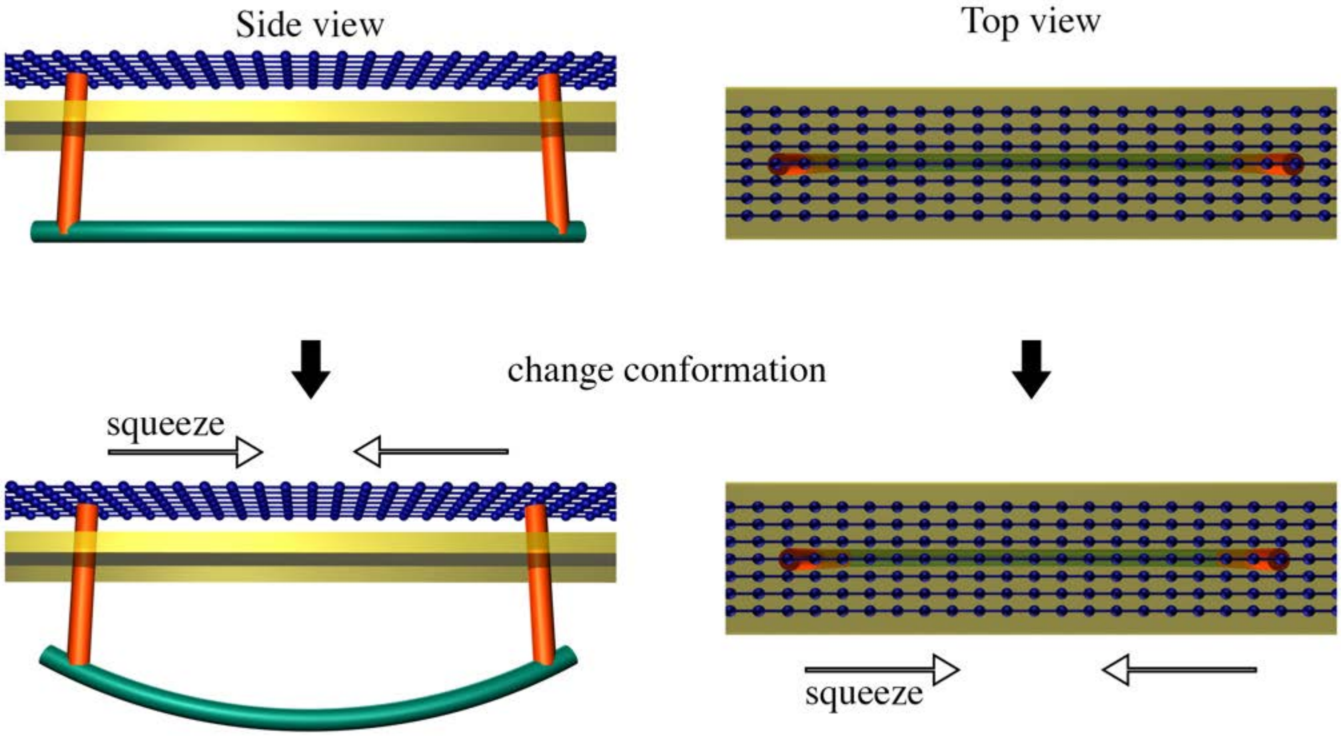

Figure S13: Schematic of a connection (orange) between the FtsZ filament (green) and the cell wall (blue). A rigid connection would allow filament bending to squeeze the local cell wall. The membrane is shown in yellow.

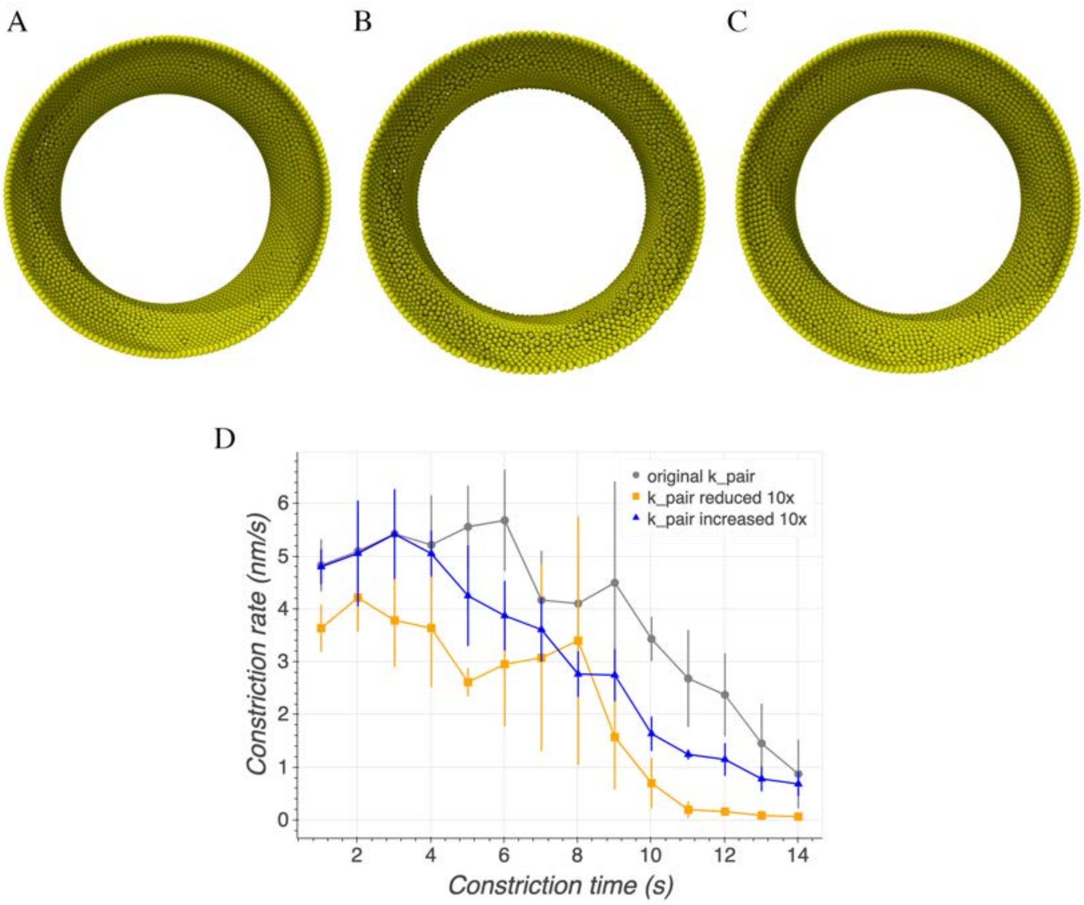

Figure S14: Varying the membrane elasticity. In these simulations, depolymerization was implemented to sustain filament sliding. (A – C) Snapshots of simulations of the filament sliding model after 14 sec of simulation time showing no apparent changes in the membrane morphology as the membrane elasticity was varied by varying the pairwise interaction force constant: (A) k_{pair} = 10 pN/nm^2, (B) k_{pair} was reduced 10 folds to 1 pN/nm^2, (C) k_{pair} was increased 10 folds to 100 pN/nm^2. (D) Time-series of the constriction rate at different values of k_{pair}.

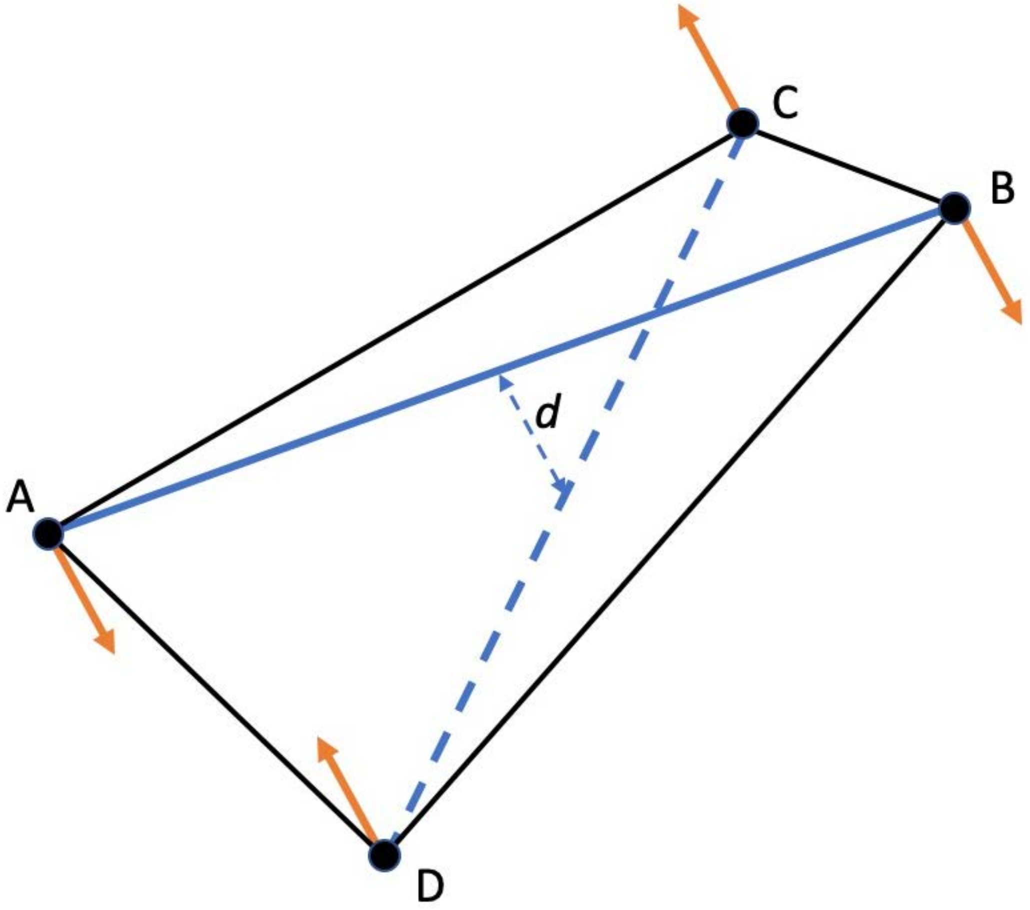

Figure S15: Schematic of constraining five pairs (solid lines in black and blue) formed by four beads A, B, C, D to the same plane. If A is paired with B, both A and B are paired with C, and both A and B are paired with D, then these four beads are confined to the same plane. If line A-B is at a distance *d* from line C-D, a force (orange) is exerted on each bead to pull the two lines into the same plane.

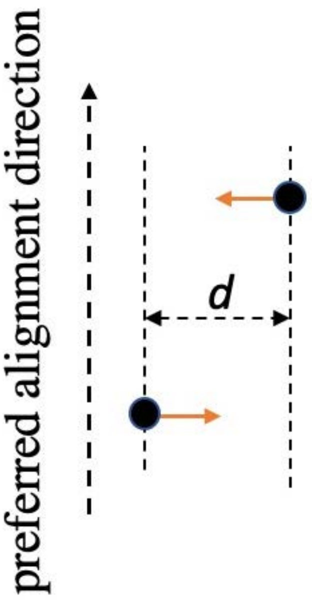

Figure S16: Schematic of restoring forces (orange arrows) exerted on two beads to align them to the preferred direction.

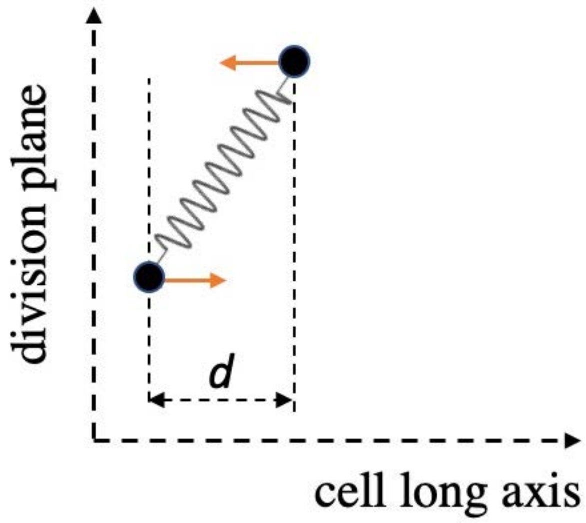

Figure S17: If a constrained spring deviated from the division plane, two forces (orange) were exerted on the two end beads to restore its preferred orientation.
